# Applying genetic technologies to combat infectious diseases in aquaculture

**DOI:** 10.1111/raq.12733

**Published:** 2022-09-05

**Authors:** Nicholas A. Robinson, Diego Robledo, Lene Sveen, Rose Ruiz Daniels, Aleksei Krasnov, Andrew Coates, Ye Hwa Jin, Luke T. Barrett, Marie Lillehammer, Anne H. Kettunen, Ben L. Phillips, Tim Dempster, Andrea Doeschl‐Wilson, Francisca Samsing, Gareth Difford, Sarah Salisbury, Bjarne Gjerde, John‐Erik Haugen, Erik Burgerhout, Binyam S. Dagnachew, Dominic Kurian, Mark D. Fast, Morten Rye, Marcela Salazar, James E. Bron, Sean J. Monaghan, Celeste Jacq, Mike Birkett, Howard I. Browman, Anne Berit Skiftesvik, David M. Fields, Erik Selander, Samantha Bui, Anna Sonesson, Stanko Skugor, Tone‐Kari Knutsdatter Østbye, Ross D. Houston

**Affiliations:** ^1^ Nofima AS Tromsø Norway; ^2^ Sustainable Aquaculture Laboratory—Temperate and Tropical (SALTT) School of BioSciences, The University of Melbourne Melbourne Victoria Australia; ^3^ The Roslin Institute and Royal (Dick) School of Veterinary Studies The University of Edinburgh Edinburgh UK; ^4^ Institute of Marine Research, Matre Research Station Matredal Norway; ^5^ Sydney School of Veterinary Science The University of Sydney Camden Australia; ^6^ Atlantic Veterinary College The University of Prince Edward Island Charlottetown Prince Edward Island Canada; ^7^ Benchmark Genetics Bergen Norway; ^8^ Institute of Aquaculture University of Stirling Stirling Scotland UK; ^9^ Blue Analytics, Kong Christian Frederiks Plass 3 Bergen Norway; ^10^ Rothamsted Research Hertfordshire UK; ^11^ Institute of Marine Research, Austevoll Research Station, Ecosystem Acoustics Group Tromsø Norway; ^12^ Bigelow Laboratory for Ocean Sciences Boothbay Maine USA; ^13^ Department of Marine Sciences University of Gothenburg Gothenburg Sweden; ^14^ Cargill Aqua Nutrition Bergen Norway

**Keywords:** gene editing, genomic selection, host resistance, sea lice, transcriptomics, white‐spot syndrome virus

## Abstract

Disease and parasitism cause major welfare, environmental and economic concerns for global aquaculture. In this review, we examine the status and potential of technologies that exploit genetic variation in host resistance to tackle this problem. We argue that there is an urgent need to improve understanding of the genetic mechanisms involved, leading to the development of tools that can be applied to boost host resistance and reduce the disease burden. We draw on two pressing global disease problems as case studies—sea lice infestations in salmonids and white spot syndrome in shrimp. We review how the latest genetic technologies can be capitalised upon to determine the mechanisms underlying inter‐ and intra‐species variation in pathogen/parasite resistance, and how the derived knowledge could be applied to boost disease resistance using selective breeding, gene editing and/or with targeted feed treatments and vaccines. Gene editing brings novel opportunities, but also implementation and dissemination challenges, and necessitates new protocols to integrate the technology into aquaculture breeding programmes. There is also an ongoing need to minimise risks of disease agents evolving to overcome genetic improvements to host resistance, and insights from epidemiological and evolutionary models of pathogen infestation in wild and cultured host populations are explored. Ethical issues around the different approaches for achieving genetic resistance are discussed. Application of genetic technologies and approaches has potential to improve fundamental knowledge of mechanisms affecting genetic resistance and provide effective pathways for implementation that could lead to more resistant aquaculture stocks, transforming global aquaculture.

## 1 INTRODUCTION

‘Blue foods’ have been highlighted as a major source of nutrients supporting the health and livelihood of many communities throughout the world.^1^ Aquaculture, currently the fastest growing food production industry, plays a key role in producing and supplying fish, shellfish and algae. However, the mass culture of animals in any environment carries with it a high risk of contracting, propagating and spreading infectious disease.^2^, ^3^ Some diseases affecting fish and shellfish can lead to 100% mortality or necessitate complete destocking. Disease prevention and treatment are necessary, but current options are often costly, ineffective and can negatively impact animal welfare, local ecosystems and product quality. For example, biosecurity is particularly challenging when animals are farmed in an open water system, and logistical difficulties in handling makes it challenging to vaccinate and treat individual animals. Here, we focus mainly on the improvement of host disease resistance which can be defined as the host's ability to reduce pathogen invasion (i.e., limiting pathogen entry into target tissues and replication).

Vaccination has proven to be an effective preventative measure boosting immunity for many major diseases affecting humans (e.g.,^4^), domestic animals and livestock including fish.^5^, ^6^, ^7^, ^8^, ^9^, ^10^, ^11^, ^12^ In some instances, total eradication of human or livestock disease has been possible through the implementation of vaccination programmes.^13^, ^14^ However, vaccines are difficult to develop against some diseases, especially against ectoparasites such as sea lice (crustacean copepods) or amoebae.^15^ Moreover, vaccination has limited success for host species with a less developed adaptive immune system such as crustaceans or molluscs.^16^ Additional tools for improving host resistance are therefore needed. In this review, we describe the use of genetic technologies that can exploit and potentially also create genetic variation in disease resistance. Such approaches can both provide a greater understanding of the natural mechanisms affecting disease resistance and can be used to boost host resistance and thereby reduce the impact of infectious diseases in aquaculture. The high fecundity of aquatic species leads to opportunities for rapid propagation and dissemination of strains with improved innate disease resistance.

Two of the most valuable global aquaculture species are salmon (with 2.6 million tonnes yearly production of Atlantic salmon, *Salmo salar*, valued at US$ 17 billion in 2019) and shrimp (with 6.2 million ton of both whiteleg shrimp *Litopenaeus vannamei* and black tiger shrimp *Penaeus monodon* valued at US$ 38.5 billion in 2019).^17^ The intensification and global nature of both Atlantic salmon and shrimp farming have led to major challenges in the form of infectious diseases. Currently, sea lice in Atlantic salmon and white spot syndrome in shrimp are two of the most pressing problems for these aquaculture industries.

Sea lice (Caligidae) are marine ectoparasitic copepods that attach to host organisms and feed on mucus, skin and blood. Most lice species have a free‐swimming planktonic larval phase (copepodid) that facilitates host‐finding, and once attached to a host, they undergo two further moults through sessile (immobile) stages before reaching mobile pre‐adult stages that can move around the host and exhibit host‐switching behaviours. They then become adults, reproducing sexually on the same host and releasing fertilised eggs into the water column.^18^, ^19^, ^20^ Sea lice have been found on numerous species of wild and farmed fish, and while the prevalence may be high, infestation densities are usually low and have relatively minor impacts on production or animal welfare (e.g.,^21^, ^22^, ^23^). The Atlantic salmon farming industry is one notable exception in which infestations can be severe and lead to poor welfare and mortality for farmed fish.^24^, ^25^, ^26^ Strict delousing regimes are used to keep lice infestation intensities low, but some delousing regimes (involving crowding, pumping and lice removal effects) can pose severe welfare risks.^27^, ^28^, ^29^ The lice larvae produced on‐farm can also infest wild salmonid populations.^30^, ^31^, ^32^, ^33^, ^34^, ^35^ Sea lice cost the Norwegian salmon sector at least 7.3 billion kroner (~US$ 810 million) per annum^36^ (up from US$ 435 million estimated for 2011^37^) and are of major concern for other salmon industries around the world. The welfare and economic impacts of louse parasitism are predicted to be even worse with higher seawater temperatures.^38^, ^39^, ^40^ *Lepeophtheirus salmonis* is the louse species currently of greatest concern in Norway, but *Caligus elongatus* has become an increasing problem (particularly in northern latitudes,^41^) and *Caligus rogercresseyi* is of great concern in Chile.^42^, ^43^, ^44^ Lice control measures entail substantial costs,^45^ with no single method proving completely effective,^46^ and moreover, lice have already evolved resistance to some methods of control (reviewed in Reference [47] e.g.^48^). Repeated reintroduction of high‐density naïve salmon hosts to sea cages is effectively a serial passage experiment that disproportionately favours the parasite's adaptation to treatments.^49^ There is an urgent need to develop new strategies to mitigate the impact of sea louse parasitism.

White spot syndrome virus (WSSV) causes devastating disease that severely impacts global shrimp aquaculture.^50^ All decapod crustaceans, including all major cultured shrimp species, are affected by WSSV, and disease outbreaks typically cause mass mortality within a few days.^51^ Elimination of the virus from the open pond systems in which shrimp are extensively grown has not been possible.^52^ Despite the lack of an adaptive immune system, stimulation of the innate immune system of shrimp by immune priming shows promise but has not yet been proven to prevent and control disease in the field.^53^, ^54^, ^55^

These two infectious diseases have a large impact on these aquaculture sectors, but similar disease problems affect all aquaculture species with devastating consequences.^56^, ^57^ Alternative measures for preventing infestation and infection are needed. Modern genetic and genomic technologies and methodologies can help us understand why some individuals, strains and species have higher inherent abilities to resist certain diseases. Such knowledge can be applied to boost the ability of farmed fish and shellfish to resist disease and reduce the burden of infectious diseases in aquaculture. Here, we will review the recent application of these technologies and explore their potential to improve the resistance of aquaculture stocks to infectious diseases in the near future.

## 2 IMPROVING HOST RESISTANCE AS A PREVENTATIVE MEASURE

Genetic improvement by selective breeding is playing a crucial role in helping aquaculture meet future demands for animal protein,^58^ and research into the genetics of disease resistance has played a major role in ensuring the health and security of aquaculture stocks.^59^ The level of host susceptibility or resistance is usually influenced by many genes each contributing small additive effects (also known as a polygenic trait). Disease resistance is usually heritable,^59^ with moderate to low heritability estimates for resistance to sea lice in Atlantic salmon (0.3^60^; 0.26–0.33^61^; 0.02–0.14^62^; 0.22^63^; 0.14–0.43^64^; 0.28^65^; 0.22–0.33^66^) and WSSV in shrimp (0.01–0.31,^67^, ^68^, ^69^, ^70^, ^71^). The potential for improving lice resistance through selective breeding is substantial as the coefficient of variation in lice count per fish is very high ranging from 60.5 to 95.0.^61^ These are within‐population estimates of heritability, but there are also very clear differences across species in susceptibility to parasitism and disease. For example, some salmonid species (e.g., coho *Oncorhynchus kisutch* and pink *Oncorhynchus gorbuscha*) have a higher innate ability to resist sea lice infection than others (e.g., Atlantic salmon *Salmo salar*, chum *Oncorhynchus keta* and rainbow trout *Oncorhynchus mykiss*).^72^, ^73^, ^74^, ^75^ Thus, there is genetic variation for resistance both within and between species that can be exploited for tackling this infectious disease.

For lice resistance, a key breeding objective trait of importance is the number of lice treatments in a production cycle (or per year) at a farm. This is determined by the treatment threshold used (the maximum number of adult female lice per fish), intensity of lice infestation and the host resistance to sea lice. A strong treatment threshold may introduce more frequent lice treatments and thus higher costs. Due to rules requiring a low threshold for delousing, it is not likely that a reduction in the number of lice treatments can be observed at a farm level over a few generations of selection. Therefore, breeding companies are reluctant to put too much weight on the lice resistance trait as this will result in reduced genetic gain for the other traits selected that pay off within a shorter time horizon. However, such a reduction is expected to be seen at a company, and in particular, at a national level. Furthermore, as direct selection for reduced number of lice treatments is not possible to apply, indirect selection through reduced lice counts per fish, or lice density obtained from a controlled challenge test, must be used instead, but this is often less efficient than direct selection.

We also need to be sure that the selection or intervention applied adequately covers interactions occurring at all relevant life stages of the pathogen and the host. In the case of salmon lice, the genetic correlation between the number of attached and adult lice counted at different times has been found to be high,^62^ and there is also a documented high genetic correlation between the number of lice per fish recorded at two subsequent challenge tests with de‐lousing after the first,^64^ as well as a high genetic correlation between the number of lice recorded on the same fish in two different times of the year (Gjerde, Pers. comm.). These results show that selection for increased resistance to lice based on lice count per fish or lice density will provide increased resistance to relevant life stages of the lice at relevant life stages of the host.

The long‐term objective of selection for increased pathogen resistance is to develop a host that the pathogen does not infect under field conditions so that the pathogen will not be able to reproduce and spread in farmed and close by wild host populations. Genetic correlations between disease resistance in a challenge test tank and under conditions in the field vary depending on the disease and are influenced by many different environmental factors. High correlations have been reported for host resistance to sea lice^62^ and to furunculosis caused by the bacterium *Aeromonas salmonidea*,^76^ and they are also likely high for host resistance to WSSV (near identical genetic ranking for survival rate among susceptible and resistant lines,^77^). This is encouraging as selection for WSSV or sea lice host resistance based on controlled challenge test data should benefit the farmers that utilise selected seedstock. On the other hand, low genetic correlations between tank and field challenges have been detected for some other parasitic diseases (e.g., amoebic gill disease in Atlantic salmon^78^).

Major challenges to genetic improvement of host disease resistance could be caused by trade‐offs between resistance, transmissibility and growth. For instance, in shrimp, there is a clear negative and thus unfavourable genetic correlation between resistance to WSSV and growth rate (−0.55 to −0.64,^69^, ^71^). In Atlantic salmon, the genetic correlation between resistance to sea lice and growth rate (−0.32 to −0.37) is also reported to be unfavourable^62^ implying that both traits need to be recorded and selected to obtain desired genetic gains for both growth rate and sea lice resistance. Another important issue that is seldom considered when selecting for improved disease resistance is possible trade‐offs between host resistance, tolerance and infectivity. Animals with high tolerance could survive longer and thus be able to transmit infectious pathogens for longer.^79^ In such instances, genetic improvement of host tolerance might not lead to beneficial epidemiological effects.

To make genetic gains in disease resistance, it would be desirable to breed from survivors to a disease if it were not possible for the survivors to transmit and propagate the disease further in the host population (i.e., hosts surviving WSSV can transmit disease). In such cases when transmission and propagation of the disease by survivors is a risk, selective breeding programmes are restricted to selection of uninfected breeding candidates (kept in a bio‐secure breeding nucleus). In the past, this selection has been based on the average performance of the infected siblings to the breeding candidates and it was not possible to capture the within‐family or Mendelian sampling component of genetic variation for sib recorded traits. Developments in genomics have enabled quantitative geneticists to exploit the broad genetic variation for host disease resistance that exists within families in these circumstances.

Marker‐assisted selection was initially touted as a technology of great promise for genetic improvement. However, because most disease traits measured to date are polygenic, and therefore single genetic markers (or single quantitative trait loci [QTL]) generally account for a small fraction of the overall genetic variation, there are few examples where the application of marker‐assisted selection has resulted in realised genetic gains larger than expected when using traditional pedigree‐based selective breeding. A notable exception where marker‐assisted selection has had a demonstrable large benefit in aquatic species is in resistance to infectious pancreatic necrosis in Atlantic salmon, where a single gene accounts for a large proportion of the overall genetic variation.^80^, ^81^, ^82^ Nonetheless, while the potential value of genomic intervention needs to be assessed for each target trait and culture environment, the latest methodologies for utilising genomic information (i.e., genomic selection,^83^) have been predicted to have high potential benefit for disease resistance and other traits that cannot be recorded on the breeding candidates,^84^ many of which are known to be polygenic in nature and difficult to directly assess on unexposed animals.

Finally, recent developments in genomic technologies provide opportunities to better understand the genetic mechanisms affecting the differences in host resistance between and within species. Not only can these technologies improve our understanding of the mechanisms involved, but they can help develop tools that could be used to reduce the impact of infectious diseases. For instance, using clustered regularly interspaced short palindromic repeat (CRISPR‐Cas) genome editing technology provides a way for making targeted changes to genes found to influence host resistance.^85^ However, the implementation of these technologies, and dissemination of genetically improved fish and shellfish for production by the industry, needs to be carefully considered to avoid potential negative environmental consequences (e.g., escapees interbreeding with wild counterparts) and counter‐adaptation by disease agents. All of these issues need to be addressed to devise an ethical and sustainable long‐term strategy for eliminating or reducing pathogen infection. As farmed hosts such as Atlantic salmon can be a major propagator, and hence, driver of disease,^34^ such a strategy should also have long‐term benefits in providing a form of ‘herd immunity’ for naturally existing populations of host species that inhabit areas close to major aquaculture environments.

## 3 BIOLOGICAL MECHANISMS AFFORDING HOST RESISTANCE

**FIGURE 1:**
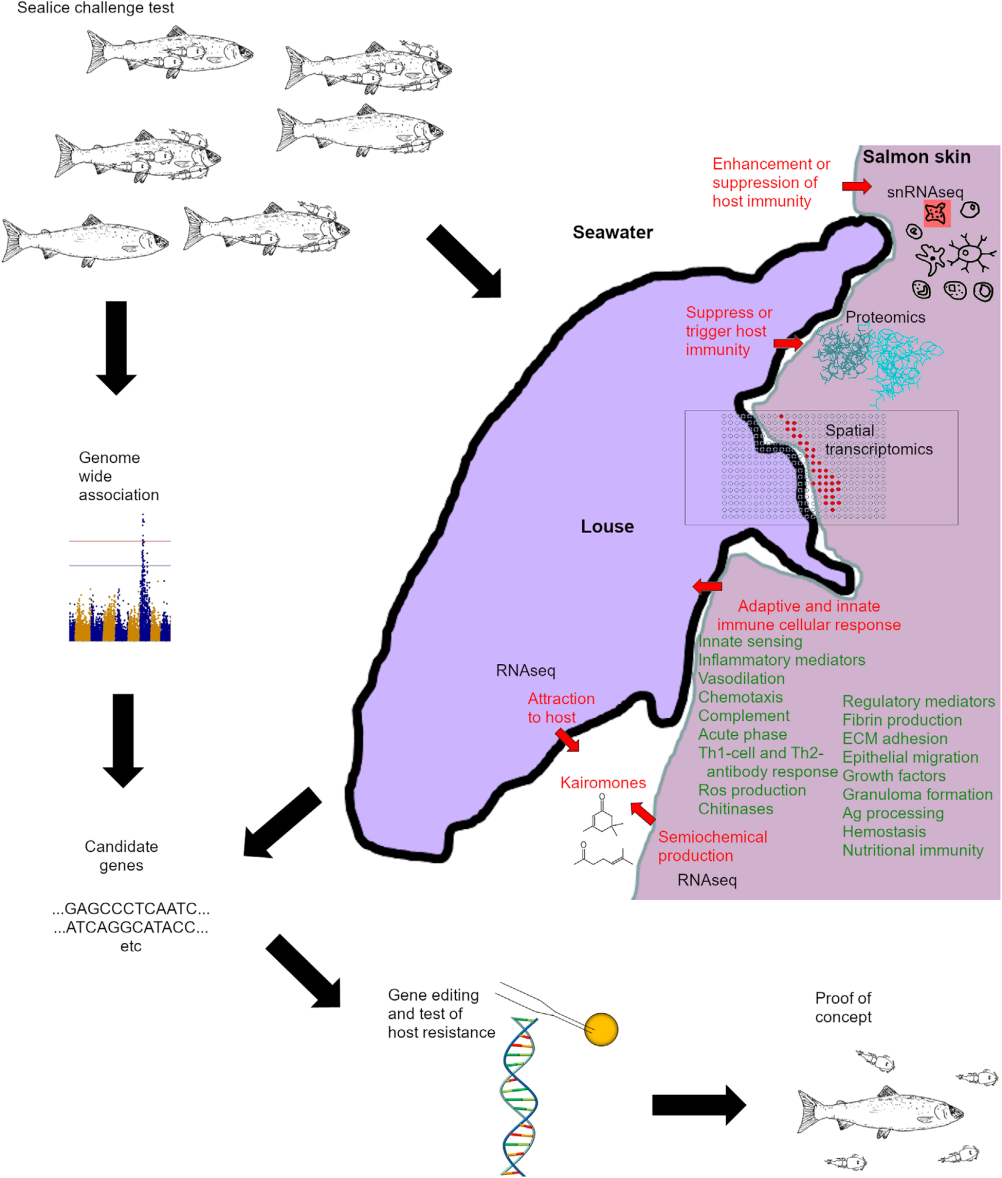
Exploring the genetic basis of mechanisms providing host resistance. Host resistance to sea lice is likely affected by environmental and dietary factors that enhance or suppress salmon immunity, the immune cellular response (adaptive and innate immune systems), kairomones that attract the lice to the host and proteins that are secreted by the louse and suppress or trigger host immunity (red text). More detailed processes and factors likely to promote host immunity in coho, pink and more resistant strains of Atlantic salmon are listed in green text. To search for genes in the host that are up‐ or down‐regulated at key time points post‐infection: (1) genome‐wide association studies can identify genes mapping to chromosomal regions associated with host resistance, (2) single nuclei RNAseq (snRNAseq) can be used to study which populations of cell types are responding in host tissue close to the interface between the salmon and the louse, (3) spatial transcriptomics and spatial proteomics can be used to map precisely where the response occurs, (4) proteomics can be used to discover interactions between host cell and lice immunomodulatory proteins (suppressing or triggering host immunity), (5) RNAseq can be used to study semiochemical production by the host and transcriptomic response of the louse in response to kairomones, and, (6) gene editing can be used to test putative genes affecting host resistance, by experimentally challenging edited and non‐edited salmon with sea lice and comparing counts of attached lice

**FIGURE 2:**
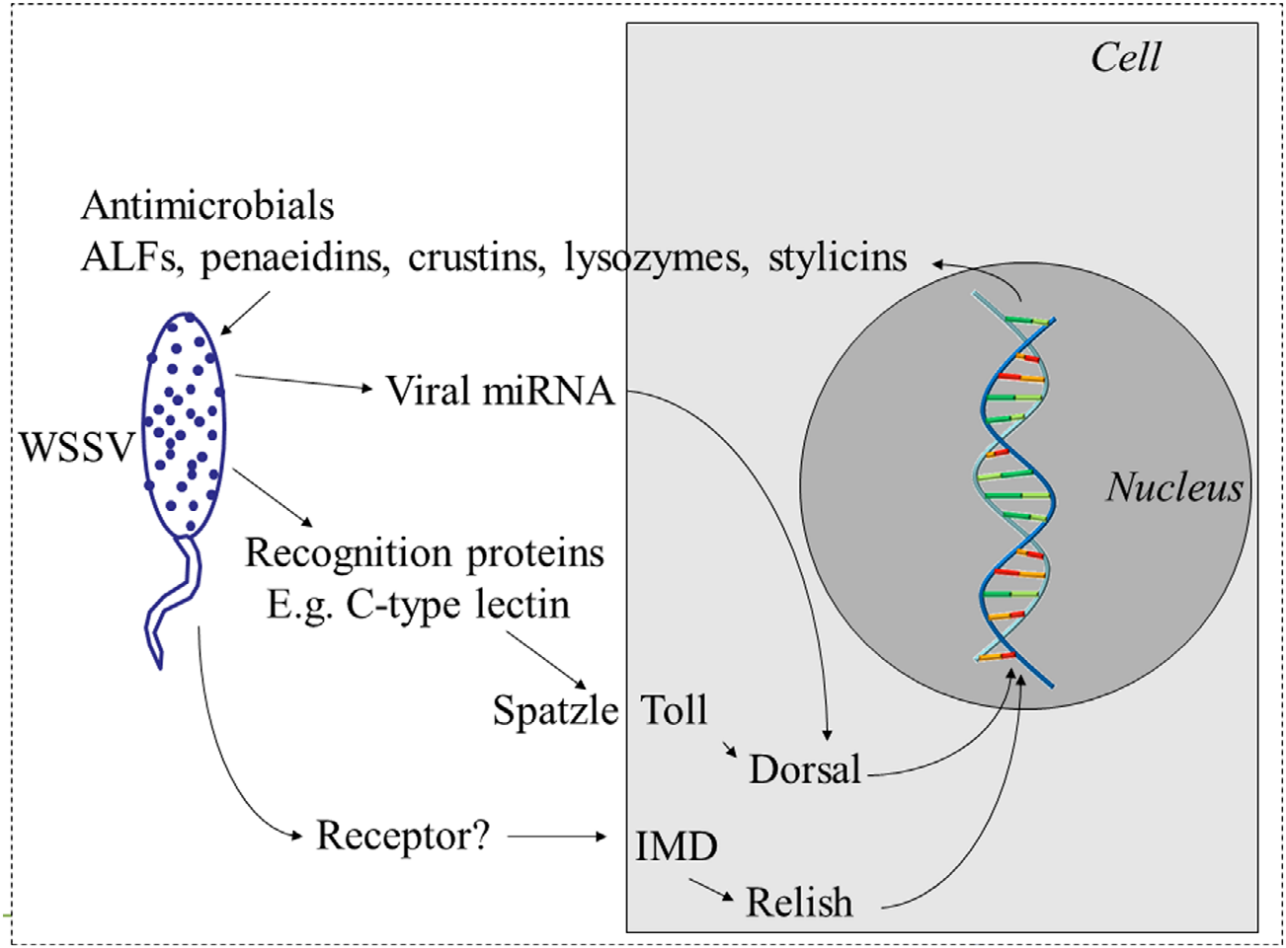
Simplified diagrammatic representation of the proposed immune response to white spot syndrome virus (WSSV) in shrimp. WSSV enters the cell using endocytic routes and induces both humoral and cellular responses from the host. Toll and immune deficiency (IMD) pathways are activated and dorsal and relsh are translocated to the nucleus inducing the expression of antimicrobial peptides that limit viral replication. C‐type lectin proteins can neutralise WSSV by binding to envelope proteins. Interestingly, WSSV is able to hijack the humoral pathways in order to facilitate its replication

Host resistance to infection and parasitism is highly complex and most likely determined by a range of responses (Figure 1), and indeed research across a range of fish species has identified broad potential mechanisms affecting host resistance.^86^ Comparatively less is known about the biology of resistance to disease in invertebrates (e.g., shrimp, Figure 2).

### 3.1 Immune and cellular response to infection and infestation

The innate immune system provides a pivotal first defence against invading pathogens and exists across invertebrate and vertebrate organisms (e.g., proposed innate immune response to WSSV in shrimp, Figure 2). Most organisms possess cellular receptors, which bind to foreign elements and differentiate self from non‐self. These pattern recognition receptors (PRRs) recognise pathogen‐associated molecular patterns (PAMPs) and damage‐associated molecular patterns (DAMPs). There are several classes of PRRS, including Toll‐like receptors (TLRs), NOD‐like receptors (NLRs) and RIG‐I‐like receptors (RLRs), each recognising distinct microbial components and directly activating immune effector cells. Whether hemocytes of crustacea, or macrophage/dendritic/B‐lymphocytes of teleosts, these phagocytic cells eliminate potential pathogens and act as the bridge to adaptive immune activation in vertebrates.

Large differences in the louse settlement success, host immune response and consequent infection of different salmon species are observed after exposure to sea lice.^73^ Although counts of settled copepodids can be initially similar in Atlantic, chum, coho, pink salmon and rainbow trout in experimental challenge tests, the intensity of infection drops in the resistant Pacific species in the first days post‐challenge.^72^ Coho and pink salmon mount a more effective response in the skin where the lice attach than Atlantic salmon, despite rapid and large‐scale immune gene activation in the skin of Atlantic salmon,^65^, ^87^, ^88^, ^89^, ^90^, ^91^, ^92^, ^93^ suggesting that resistance is determined by the character rather than magnitude of response. Resistance to the parasite seems a highly complex biological process. Within 24 h of parasite settlement coho salmon exhibit a multifocal inflammatory granulocytic infiltration, associated with a pronounced non‐specific epithelial hyperplasia and melanin deposition over the coming days. Epidermal hyperplasia is intensified and accompanied by eosinophilic granular cell infiltration by 7 days and encapsulation of the parasite by 10 days post infestation, with limited pathology thereafter.^94^

In contrast, this response is not observed in pink salmon (also highly resistant), which instead develop a rapid inflammatory response.^74^, ^95^ Tadiso et al.^93^ found transient increases of mannose‐binding receptors and C‐type lectins in Atlantic salmon skin, yet these were minor and largely absent after 3 days post‐infection. In coho salmon, local and systemic induction of chitin, mannose and other non‐self PRRs initiate the acute inflammation preceding louse encapsulation and rejection. Similarly, whereas acute phase response genes, extracellular matrix (ECM) and tissue remodelling matrix metalloproteinases (MMPs) are all down‐regulated in this time frame for Atlantic salmon, they are upregulated in Coho salmon.^96^, ^97^, ^98^

Although Atlantic salmon develops a rapid local and systemic transcriptomic response to *L. salmonis*, this does not result in substantial levels of protection in most individuals,^93^ and immunomodulation by the parasite has been suggested to play an important role in the early stages of louse attachment.^74^, ^99^, ^100^, ^101^, ^102^, ^103^, ^104^, ^105^, ^106^ A range of anti‐host proteins, including proteolytic enzymes and prostanoids, are secreted by a variety of louse exocrine glands and pass to host tissues from the point of louse attachment. These serve to suppress host immune responses during the initial stages of attachment^107^, ^108^, ^109^ and different protease profiles have been identified between excretory/secretory (E/S) products from pre‐adult and adult *L*. *salmonis*.^110^ Host‐ and context‐specific expression of genes in the louse (e.g.,^111^, ^112^, ^113^) may also provide a key to understanding species‐specific levels of host resistance.

Similarly, infection with WSSV in shrimp results in a complex range of host‐pathogen interactions in humoral and cellular pathways (Figure 2). The virus may enter the cell by several endocytic routes, and after this Toll and immune deficiency (IMD) signalling pathways are activated resulting in the up‐regulation of the NF‐kB transcription factors Dorsal, Relish and AP‐1 that increase the expression of antimicrobial peptides (AMP).^114^, ^115^ To date, several classes of AMPs or effectors have been identified in shrimp: Penaeidins (PEN), Crustins (Cru), anti‐LPS‐factors (ALF), C‐type lectins (CTL), Lysozymes (Lyz), and thioester‐containing proteins (TEP).^116^, ^117^

WSSV has also been shown to immunomodulate the shrimp response. Inhibition of the NF‐kB signalling is achieved by encoded microRNAs (WSSV‐miR‐N13 and WSSV‐miR‐N23), which can target Dorsal.^118^ WSSV also modulates the Toll dorsal pathway at different levels, for example, the WSSV449 protein; homologous to tube insect protein, can activate NF‐kB to trigger promoters of viral genes such as wsv069, wsv303 and wsv371.^119^

Another pathway relevant to the immune responses of both invertebrates and vertebrates is the JAK/STAT pathway. In shrimp, activation of the JAK/STAT pathway by bacterial challenge increases expression of AMPS *ALF‐A1*, *ALF‐C1*, *ALF‐C2*, *CruΙ‐1* and *CruΙ‐5*.^120^ Upon WSSV infection in shrimp the expression of phosphorylated STAT is upregulated, and STAT is translocated to the nucleus. However, the binding motif of STAT binds to the WSSV 1E1 promoter region facilitating, instead of inhibiting, viral replication.^121^

Apoptosis also plays a critical role in vertebrate and invertebrate defence against viral pathogens. In insects, apoptosis has an antiviral action to control viral pathogens,^122^ in shrimps, however, the role of apoptosis during WSSV infection is not clear. The viral accommodation theory says that apoptosis can be used by the virus to promote infection and that reduced rates of cell death can be the cause of the reduction of viral pathogenicity in shrimps.^123^ However, other reports have shown that cells displaying nuclear condensation and fragmentation characteristics of apoptosis did not contain WSSV virions, whereas those containing WSSV virions were not apoptotic,^124^, ^125^ suggesting that WSSV is able to inhibit apoptosis of infected cells. Several mechanisms have been identified, with caspases as the main target of viral proteins. The viral protein AAP‐1, encoded by WSSV449, contains two putative caspase‐9 cleavage sites, VETD233G and LEHD303G, as well as a caspase‐3 cleavage site, DEVD272G. WSV222, which is an E3 ubiquitin ligase, blocks apoptosis through the ubiquitin‐mediated degradation of the shrimp pro‐apoptotic TSL protein.^126^

**FIGURE 3:**
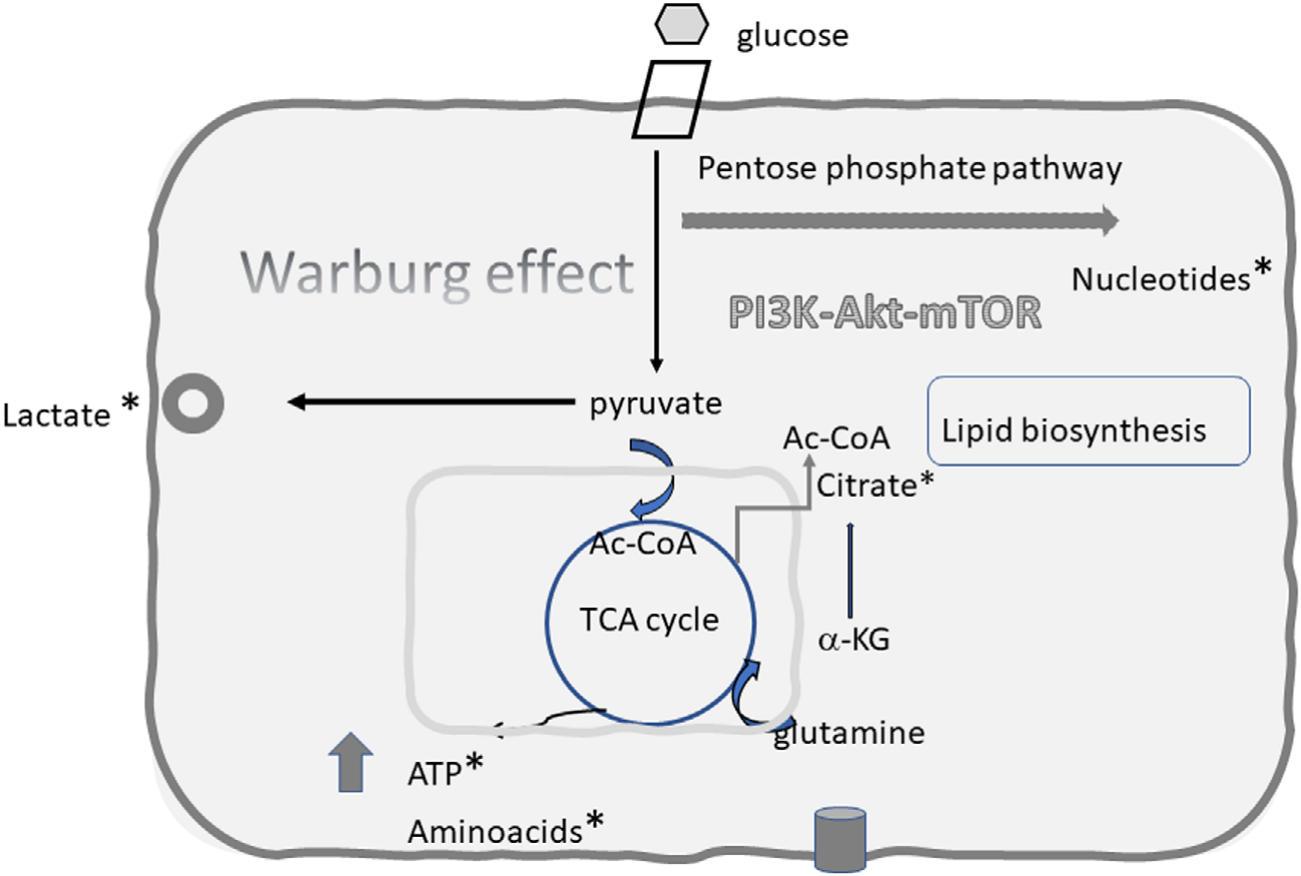
Summary of metabolic effects during early (6–12 h) white spot syndrome virus (WSSV) infection. WSSV triggers aerobic glycolysis (Warburg effect) via activation of the PI3K‐Akt‐ mTOR pathway. The hallmark of aerobic glycolysis is the high levels of glucose consumption and lactate production. However other metabolic pathways are also enhanced, including the pentose phosphate pathway (PPP), the lipid metabolism pathway and the glutamine metabolism pathways. These metabolic changes support the high energy requirements of viral replication

A holistic approach using ‘omic’ technologies (including metabolomics^127^ and glycomics^128^ which are not detailed in this review) could help elucidate the complex interaction between WSSV and the crustacean host. WSSV has been shown not only to modulate the hosts immune response, but also to interfere with shrimp cell metabolism, being the first invertebrate virus known to induce aerobic glycolysis in infected cells (Warburg effect)^129^ via the PI3K‐Akt‐ mTOR pathway, which is activated upon WSSV infection. Metabolic reprograming induced by WSSV involves glycolysis, the tricarboxylic acid (TCA) cycle, glutaminolysis and lipid metabolism^130^ (Figure 3). Su et al.^131^ demonstrated that pre‐treatment of shrimp with a PI3K inhibitor was associated with a decrease in WSSV gene expression and copy number, indicating the WSSV‐induced Warburg effect is important for successful viral replication. Hypoxia‐inducible factor‐1 (HIF‐1), a transcriptional factor that regulates the expression of several glycolytic genes, is also involved in the metabolic reprogramming induced by WSSV. HIF‐1 silencing in WSSV‐infected shrimps blocks the upregulation of the glycolytic enzymes HK, PFK and PK and the expression and activity of LDH, increasing survival of the infected animals.^132^

Immune training or priming using heat‐killed pathogens, heat shock proteins or poly(I.C) mimicking double‐stranded viral RNA can trigger strong effector immune responses in invertebrates^53^, ^55^, ^133^, ^134^, ^135^ and knowledge about specific gene products with potential high efficacy for immune priming resulting in disease resistance is needed.

### 3.2 Semiochemical production

Another important consideration is the question of what attracts parasites to a particular host or deters parasites from infecting other hosts or non‐hosts. Semiochemicals are chemical substances released by one organism that affect the behaviour of other organisms. Semiochemicals that attract another species and harm the emitting host species are known as kairomones whereas those that deter the other species and benefit the emitting host species are known as allomones. Copepodids suspended in the water column detect and show a positive rheotactic response to nearby hosts guided by chemical and mechano‐sensory clues.^136^, ^137^, ^138^, ^139^, ^140^ Host‐derived kairomones (reviewed in Reference [141]) play a key role in attracting lice at close distances.^138^ Silencing of chemoreceptors in copepodids interferes with recognition of the host.^142^, ^143^ *L. salmonis* is highly host‐specific, largely infecting fish of *Salmo* and *Oncorhynchus* genera with different rates of success, whereas *C. elongatus* parasitizes more than 80 marine fish species.^144^ This suggests a combination of species‐specific kairomones that attract *L. salmonis* to susceptible species (*Salmo* spp.) and allomones that deter *L. salmonis* from the more resistant species including *Oncorhynchus* spp.^141^ The evidence also suggests that *C. elongatus* attracting kairomones are produced by many species.

Behavioural tests (directional movement of salmon lice and electrophysiology) show that lice are attracted to water conditioned with salmon and that the effect is associated with the volatile fraction.^145^ Specific compounds such as isophorone (3,5,5‐trimethylcyclohex‐2‐en‐1‐one) and sulcatone (6‐methyl‐5‐hepten‐2‐one) have been identified as candidate lice attracting kairomones for Atlantic salmon.^140^, ^146^

In addition to variation in attractants in the water among and within species, there is individual variation among hosts with respect to the production of certain compounds in the mucus. Volatile organic compounds including 1‐octenol‐3‐ol, 1‐penten‐3‐ol and sulcatone in the mucus of Atlantic salmon have been found to be associated with increased sessile lice count.^147^ These compounds are secondary lipid oxidation products which have been linked to host identification in biting insects such as mosquitoes and midges.^148^, ^149^, ^150^ The concentration of these compounds in mucus differs between different families of Atlantic salmon (37%–58%) fed the same diet and is associated with higher lice counts in individuals.^151^

Targeted chemical analysis (e.g.,^152^) of such mucous compounds may allow identification of phenotypes that can be used in selective breeding. More information is, however, needed on the genetic basis and heritability of the production of semiochemicals for this purpose. Using genomic or proteomic data to dissect susceptibility to louse infestation into several underlying and more objective component phenotypes could potentially offer more specific information to complement and improve the accuracy of selection. For instance, semiochemicals that are exuded from the skin in the mucus could be sampled non‐invasively from the host and could have potential as markers for selection.

## 4 EXPLORING THE GENETIC FACTORS UNDERLYING HOST RESISTANCE

### 4.1 Genomic tool application

Host–parasite and host–viral interactions are extremely complex, and their study requires a holistic approach involving the use of technologies that investigate different aspects of the interaction (Figure 1 and Table 1). Knowledge about the specific genes and mechanisms involved in providing greater disease resistance are needed, particularly for potential gene editing applications downstream. In this section, we provide an overview of the different genomic technologies that can elucidate different components of host–pathogen interactions. While the generation of multiomics datasets is likely to help elucidate host–pathogen interactions and other biological processes, extracting meaningful information from these types of datasets will require appropriate integration methods. The recent explosion of genomics has led to the development of multiple methods for the integration of different data, based on very different approaches ranging from k‐means clustering or linear models to complex Bayesian models.^175^ The choice of methods to use for the most common integration challenges will depend on the types of data and on the availability of already tailored methods. For instance, single‐cell RNA‐seq can be easily integrated with single‐cell ATAC‐seq or spatial transcriptomics using the freely available R package Seurat.^176^ The usefulness of these complex datasets will depend on our understanding of gene/protein function. Mapping protein interactions will also provide fundamental information that should help improve our understanding of the relationship between each aquaculture species and its pathogens.^177^

**TABLE 1:** Examples of ‐omic technologies with potential applications for exploring the genetic basis of host resistance

‐Omic technology	Example	Application	Relevance in the study of disease resistance	Key references
Genomics	GWAS	Detection of genomic regions and allelic variants associated with phenotypic variation (e.g., disease resistance)	Fine mapping of loci associated with host resistance providing candidates for further testing (and potentially enabling marker‐assisted selection)	[153]
	Gene editing	Study of gene function	Identification and or confirmation of relevant /causative genes involved in disease resistance	[154]
	Genome‐wide CRISPR knock‐out screens	Assessment of the impact of all the genes in a genome on a phenotype	Unbiased identification of genes involved in disease resistance	[155]
Transcriptomics	RNAseq	Transcriptomic differences between tissue samples	Changes in the transcriptome of a tissue sample in response to infection.	[156, 157]
	scRNA‐seq	Transcriptomes measured at single‐cell resolution	Detects changes in cell populations and gene expression of each cell type in response to infections	[158, 159]
	snRNA‐seq	Similar to scRNAseq but using nuclei instead of whole cells	As per scRNAseq	[160, 161]
	Spatial transcriptomics	Transcripts mapped and quantified to their position in a tissue section	Expression changes at spatial resolution in the tissue section in response to infection	[162, 163]
	Perturbation screens	Combination of pooled CRISPR screens and scRNAseq to study the function of multiple genes	Transcriptomic changes induced by the absence or up‐regulation of a gene of interest at cellular resolution	[164]
Biomolecule omics	Mass spectrometry	Identifying proteins, carbohydrates, glycans, glycoproteins and metabolites produced by host.	Identify semiochemicals produced by host and parasite–host protein interactions	[165]
	Spatial proteomics	Similar to spatial transcriptomics but for mass spectrometry identification of proteins	How are protein interactions differing in areas of the tissue section responding to infection	[166]
Epigenomics	CHIA‐PET or HIC	Generation of genome‐wide chromatin interaction maps	Identify genomic regions and sequences showing differential gene regulation	[167, 168]
	ATACseq and scATAC‐seq	Map chromatin accessibility (potentially at single‐cell resolution)	Identify differences in chromatin accessibility are associated with disease resistance.	[169, 170]
	CHIPseq	Genome‐wide identification of the binding sites of DNA‐associated proteins	Identify differences in DNA binding of specific transcription factors and other chromatin‐associated proteins associated with disease resistance	[171]
	WGBS	Methylation status at each cytosine base across the genome	Identify differences in DNA methylation associated with disease resistance	[172, 173]
	RRBS	As for WGBS but assaying only genomic regions with high CpG content	As for WGBS	[174]

Abbreviations: GWAS, genome‐wide association studies; scRNA, Single‐cell RNA sequencing; snRNA, Single‐nuclei RNA sequencing; CHIA‐PET, chromatin interaction analysis with paired‐end‐tag‐sequencing; ATACseq, assay for transposase‐accessible chromatin with high throughput sequencing; CHIPseq, chromatin immunoprecipitation sequencing; WGBS, whole‐genome bisulphite sequencing; RRBS, reduced representation bisulfite sequencing.

#### 4.1.1 Genome‐wide association of marker tests

Genome‐wide association studies (GWAS) have probably been the most important contributor to the identification of genes affecting disease resistance so far (Tables 1 and 2). These approaches detect specific regions of the genome associated with increased resistance to a disease (QTL), and rely on the existence of genetic variation in resistance among the animals of a population. Resistance is typically measured as morbidity or mortality/survival via controlled disease challenges or natural outbreaks, although more complex phenotypes such as time to death or pathogen load are also frequently used. The final goal is to detect major QTL that can be selected for or against in aquaculture breeding programmes using associated genetic markers (marker‐assisted selection). However, QTLs with large effects on the trait of interest are infrequent, and resistance to most diseases is polygenic in nature.^242^

**TABLE 2:** Published application of single nucleotide polymorphism (SNP) genome‐wide association studies (GWAS) and quantitative trait loci (QTL) scans to explore the genetic basis of host resistance for species in aquaculture for food production

**Atlantic salmon**
Infectious salmon anaemia^178^, ^179^
Pancreas disease^180^, ^181^
Piscine myocarditis virus^182^, ^183^, ^184^
Amoebic gill disease^185^, ^186^, ^187^
Sea lice^65^, ^188^, ^189^
Infectious pancreatic necrosis^81^, ^154^, ^190^, ^191^
*Piscirickettsia salmonis* ^192^
**Coho salmon**
*Piscirickettsia salmonis* ^193^
**Common carp**
*Aeromonas hydrophila* ^194^
Koi herpesvirus^195^, ^196^, ^197^
**Rohu carp**
*Aeromonas hydrophila* ^198^
**Rainbow trout**
*Aeromonas salmonicida* ^199^
*Vibrio anguillarum* ^200^
*Ichthyophthirius multifiliis* ^201^
*Piscirickettsia salmonis* ^202^
Infectious pancreatic necrosis^203^
Columnaris^204^
Coldwater bacterial disease^205^, ^206^, ^207^, ^208^
Infectious pancreatic necrosis^203^, ^209^
Viral haemorrhagic septicaemia virus^210^
**Nile tilapia**
Tilapia lake virus^211^
**Red tilapia**
*Streptococcus agalactiae* ^212^
**European sea bass**
Nervous necrosis virus^213^, ^214^
Vibriosis^215^
**Asian sea bass**
Nervous necrosis virus^216^, ^217^
**Red spotted grouper**
Nervous necrosis virus^218^
**Yellowtail (Seriola quinqueradiata)**
Benedenia disease^219^, ^220^, ^221^
**Pacu**
*Aeromonas hydrophila* ^222^
**Catfish**
*Aeromonas hydrophila* ^223^
Enteric septicemia^224^, ^225^, ^226^
Columnaris disease^227^
**Yellow croaker**
*Cryptocaryon irritans* ^228^
*Pseudomonas plecoglossicida* ^229^
**Gilthead seabream**
Phytobacteriosis^230^
*Sparicotyle chrysophrii* ^231^
**Japanese flounder**
*Vibrio anguillarum* ^232^
**Turbot**
*Aeromonas salmonicida* ^233^
*Philasterides dicentrarchi* ^234^, ^235^
**Red sea bream**
Iridoviral disease^236^
**Black tiger shimp**
White spot syndrome virus^237^
**Pacific oyster**
*Vibrio alginolyticus* ^238^
Ostreid herpesvirus^239^
**European flat oyster**
*Bonamia ostreae* ^240^
**Hard clam**
Quahog parasite unknown disease^241^

*Note*: Disease names or pathogens are listed below each host species (host species in bold). Commonly used names are included.

Although QTL for ‘lice‐attracting salmon’ have been found (www.aquagen.no,^65^), lice resistance measured as lice count and lice density seems to be polygenic.^243^ Efforts to dissect the genetic mechanisms behind salmon lice resistance have yielded three chromosomal regions in which there are genes explaining between 7% and 13% of the genetic variation in resistance to *C*. *rogercresseyi* in Atlantic salmon in Chile.^65^ The effect of individual QTL on resistance is relatively small, and therefore genomic selection is preferred over marker‐assisted selection. Knockout or up‐regulation of the causative genes via genome engineering (e.g., gene editing) might provide greater resistance than the causative variants themselves. Gene expression data and knowledge from other experiments, some comparing the transcriptome of resistant and susceptible Atlantic salmon (e.g.,^87^, ^90^, ^93^), could be drawn upon to identify candidate genes and potential causative mutations associated with QTL. These could then be edited using CRISPR/Cas9 to generate beneficial variation de novo (see below), potentially with a larger effect than the detected QTL.

GWAS has detected several QTL associated with WSSV resistance (time to death) in *P. monodon* ^237^ and identified nearby mapping genes that could potentially influence innate immunity. The estimated size of the effects of these QTL on resistance was relatively small. Some of the single nucleotide polymorphisms (SNPs) mapping to these regions occurred in genes affecting the development and function of the innate immune system such as those in the ubiquitin‐proteosome pathway, affecting lymphocyte cell function, heat shock proteins, the TOLL pathway, protein kinase signal transduction pathways, mRNA binding proteins and lectins. Genome scans of broader shrimp populations utilising denser sets of SNP markers mapping to the QTL regions of interest can be used to verify and narrow the region searched for the occurrence of putative causative genes. Further work is needed to study the response of these and other candidate genes to WSSV infection in susceptible and resistant shrimp and to understand whether the same mechanisms might be involved in other penaeid shrimp such as *L. vannamei*. GWAS has been used in this way as a ‘first pass screen’ to help target future research efforts.

#### 4.1.2 Single‐cell RNA sequencing

Sequence count data, derived from the application of RNA‐sequencing (Table 1), has been used to compare the host gene expression response to infection in highly susceptible and resistant individuals for many aquatic diseases of concern to aquaculture (Table 3). In contrast to bulk RNA‐seq, single‐cell sequencing provides sequences of the transcriptomes of individual cells. Single‐cell sequencing has the resolution to identify cell types and transcript changes of interest during a disease challenge that would otherwise be indistinguishable with bulk sequencing^502^ and since being named method of the year by Nature in 2013,^158^ single‐cell RNA sequencing (scRNAseq) has provided novel insights into infectious disease response and host‐pathogen interactions (reviewed in Reference [159]). The most widely used platforms (e.g., 10× Genomics Technology) use microfluidics to capture individual cells with uniquely barcoded beads, each of which are covered with unique molecular identifiers that tag each individual RNA molecule within a cell. This combination of barcodes allows tracing of each RNA molecule to its cell of origin.^160^

**TABLE 3:** Published application of RNA‐seq to explore the genetic basis of host resistance for species in aquaculture for food production

**European sea bass**
*Ceratothoa oestroides* ^244^
Nodavirus^245^, ^246^, ^247^
*Vibrio anguillarum* ^248^, ^249^
**Japanese sea bass**
*Vibrio harveyi* ^250^
**Largemouth bass**
*Micropterus salmoides* rhabdovirus^251^
**Blunt snout bream**
*Aeromonas hydrophila* ^252^
**Gilthead sea bream**
*Sparicotyle chrysophrii* ^253^
**Red sea bream**
*Vibrio anguillarum* ^254^
**Sea bream**
Nervous necrosis virus^255^
**Black carp**
*Aeromonas hydrophila* ^256^
**Catla carp**
*Edwardsiella tarda* ^257^
**Common carp**
*Aeromonas hydrophila* ^258^
*Aphanomyces invadans* ^259^
Cyprinid herpesvirus 3^260^, ^261^
Koi herpesvirus^262^
Spring viraemia of carp virus^263^
**Gibel carp**
Cyprinid herpesvirus 2^264^, ^265^
Liver myxobolosis^266^, ^267^
**Grass carp**
*Aeromonas hydrophila* ^268^, ^269^
Grass carp reovirus^270^, ^271^, ^272^, ^273^, ^274^
*Ichthyophthirius multifiliis* ^275^
*Vibrio mimicus* ^276^
**Rohu carp**
*Aphanomyces invadans* ^277^
*Aeromonas hydrophila* ^278^
**Silver carp**
*Microcystis aeruginosa* ^279^
**African catfish**
*Aeromonas veronii* ^280^
**Channel catfish**
Channel catfish virus^281^
*Edwardsiella ictaluri* ^282^, ^283^, ^284^, ^285^, ^286^, ^287^, ^288^
*Flavobacterium columnare* ^284^, ^285^, ^286^, ^287^, ^288^, ^289^, ^290^, ^291^, ^292^, ^293^, ^294^, ^295^
Enteric septicemia^296^, ^297^
*Yersinia ruckeri* ^298^
**Ussuri catfish**
*Aeromonas veronii* ^299^
**Yellow catfish**
*Edwardsiella ictaluri* ^300^
**Cobia**
*Streptococcus dysgalactiae* ^301^
**Yellow croaker**
*Pseudomonas plecoglossicida* ^229^, ^302^, ^303^, ^304^, ^305^
**European eel**
*Aeromonas hydrophila* ^306^
Rana grylio virus and the *Herpesvirus anguillae* ^307^
*Anguillicola crassus* ^308^
**Japanese eels**
*Anguillicola crassus* ^308^
**Olive flounder**
*Edwardsiella tarda* ^309^, ^310^
Megalocytivirus^310^
*Hirame novirhabdovirus* ^311^, ^312^
Infectious haematopoietic necrosis virus^312^
*Listonella anguillarum* ^313^
Lymphocystis disease virus^314^
Megalocytivirus^315^
Viral haemorrhagic septicaemia virus^312^, ^316^, ^317^, ^318^, ^319^, ^320^, ^321^
**Gibelcarp**
cyprinid herpesvirus 2^322^
**Fat greenling**
*Vibrio harveyi* ^323^
**Brown‐Marbled Grouper**
*Epinephelus fuscoguttatus* ^324^
**Giant grouper**
Spotted knifejaw iridovirus^325^
**Malabar grouper**
Nervous necrosis virus^326^
**Kelp grouper**
Nervous necrosis virus^327^
**Orange‐spotted grouper**
Iridoviruses^328^
Nervous necrosis virus^329^, ^330^
*Pseudomonas plecoglossicida* ^331^, ^332^, ^333^, ^334^, ^335^, ^336^, ^337^
**Red spotted grouper**
Nervous necrosis virus^338^, ^339^, ^340^
**Tiger grouper**
*Spatholobus suberectus*, *Phellodendron amurense*, or *Eclipta prostrata* ^341^
**Yellowtail Kingfish**
Gut enteritis^342^
**Lumpfish**
*Vibrio anguillarum* ^343^
**Mandarin fish**
Infectious spleen and kidney necrosis virus^344^
**Fathead Minnow**
*Yersinia ruckeri* ^345^
**Rare Minnow**
Grass carp reovirus^346^
**Pacu**
*Aeromonas hydrophila* ^347^
**Yellow perch**
*Apophallus brevis* ^348^
**Black rockfish**
*Photobacterium damselae* ^349^
**Atlantic salmon**
Infectious salmon anaemia virus^178^, ^350^, ^351^, ^352^, ^353^, ^354^, ^355^, ^356^
Amoebic gill disease^185^, ^357^, ^358^, ^359^
Salmon and sea lice^65^, ^87^, ^351^, ^360^, ^361^, ^362^
*Aeromonas salmonicida* ^363^
Infectious haematopoietic necrosis virus^364^
Infectious pancreatic necrosis virus^365^, ^366^
*Pilchard orthomyxovirus* ^367^
*Piscirickettsia salmonis* ^192^, ^368^, ^369^, ^370^
Pancreas disease^180^, ^371^, ^372^, ^373^
*Saprolegnia parasitica* ^374^
**Sockeye salmon**
Piscine reovirus and infectious haematopoietic necrosis virus^375^
Rhabdovirus^376^
**Asian seabass**
Scale drop disease virus and lates calcarifer Herpes virus^377^
Viral nervous necrosis^378^, ^379^
**Hybrid snakehead**
*Aeromonas schubertii* ^380^
**Snakehead**
Vesiculovirus^381^, ^382^
**Half‐smooth tongue sole**
*Vibrio anguillarum* ^383^
**Senegalese sole**
Nervous necrosis^384^
**Steelhead**
*Ceratonova shasta* ^385^
**Amur Sturgeon**
*Mycobacterium Marinum* ^386^
*Yersinia ruckeri* ^387^, ^388^
**Nile tilapia**
*Saprolegnia parasitica* ^374^, ^389^
*Francisella noatunensis* ^390^
Meningoencephalitis^391^
*Streptococcus agalactiae* ^392^, ^393^, ^394^, ^395^, ^396^, ^397^, ^398^, ^399^, ^400^, ^401^
*Streptococcus iniae* ^402^
Tilapia lake virus^403^, ^404^, ^405^
**Brown trout**
Proliferative darkening syndrome^406^
*Tetracapsuloides bryosalmonae* ^407^
**Rainbow trout**
infectious haematopoietic necrosis virus^408^, ^409^
*Yersinia ruckeri* ^410^
*Aeromonas salmonicida* and *Ceratomyxa shasta* ^409^
*Flavobacterium psychrophilum* ^411^, ^412^, ^413^
Glochidia^414^
*Ichthyophthirius multifiliis* ^415^, ^416^, ^417^
Infectious pancreatic necrosis virus^418^
*Piscirickettsia salmonis* ^419^, ^420^
Proliferative kidney disease^421^
*Vibrio anguillarum* ^422^
*Ceratonova shasta* ^423^
**Steelhead trout**
*Ceratonova shasta* ^423^
**Turbot**
*Edwardsiella piscicida* ^424^
*Enteromyxum scophthalmi* ^425^, ^426^
*Vibrio anguillarum* ^427^
**Mitten Crab**
*Micrococcus luteus*, *Vibrio alginolyticus* and *Pichia pastoris* ^428^
**Mud Crab**
*Vibrio parahemolyticus* ^429^
White spot syndrome virus^430^, ^431^
**Australian red claw crayfish**
*Aeromonas veronii* ^432^
**Red swamp crayfish**
Infectious hypodermal and haematopoietic necrosis virus^433^
*Aeromonas hydrophila* ^434^
*Vibrio cholerae* ^435^
WSSV and *Aeromonas hydrophila* ^436^
**Far eastern mussel**
*Vibrio alginnolyficus* ^437^
**Bannana shrimp**
Hepatopancreatic parvo‐like virus^438^
White spot syndrome virus^439^
**Black tiger shrimp**
Acute hepatopancreatic necrosis disease^440^, ^441^
Decapod iridescent virus 1^442^
Hepatopancreatic necrosis disease^443^
White spot syndrome virus^444^
**Chinese grass shrimp**
*Tachaea chinensis* ^445^
**Chinese mitten crab**
White hepatopancreas syndrome^446^
**Fairy Shrimp**
Bacterial black disease^447^
**Giant fresh water prawn**
*Enterobacter cloacae* ^448^
Nodavirus^449^
*Vibrio parahaemolyticus* ^450^
White spot syndrome virus^451^
**Kuruma shrimp**
*Vibrio alginolyticus* ^452^
White spot syndrome virus^453^, ^454^, ^455^
**Whiteleg shrimp**
*Spiroplasma eriocheiris* ^456^
Taura syndrome virus^457^, ^458^
*Vibrio parahaemolyticus* ^459^, ^460^, ^461^, ^462^, ^463^, ^464^, ^465^
White spot syndrome virus^466^, ^467^, ^468^, ^469^, ^470^, ^471^, ^472^, ^473^, ^474^
**Manila clam**
*Perkinsus olseni* ^475^, ^476^
*Vibrio anguillarum* ^477^
*Vibrio tapetis* ^478^
Brown muscle disease^479^
**Surf clams**
*Vibrio* spp.^480^
**Pacific oyster**
*Vibrio splendidus* ^481^, ^482^
Norovirus^483^
Ostreid herpesvirus^135^, ^484^, ^485^
*Staphylococcus aureus* ^482^
**Pearl oyster**
*Vibrio alginolyticus* ^486^
**Blackfoot paua**
Abalone viral ganglioneuritis^487^
**Coloured abalone**
*Haliotid herpesvirus‐1* ^488^
Malacoherpesviruses^489^
**Chinese razor clam**
*Vibrio parahaemolyticus* ^490^, ^491^
**Spotted hard clam**
*Vibrio parahaemolyticus* ^492^
**Cupped oyster**
*Ostreid herpesvirus 1* ^493^
**Eastern oyster**
*Perkinsus marinus* ^494^, ^495^, ^496^
**American oysters**
*Perkinsus marinus* ^497^
Roseovarius oyster disease^498^
**Japanese sea cucumber**
*Vibrio splendidus* ^499^
**Sea urchin**
Spotting disease^500^
*Vibrio* sp.^501^

*Note*: Disease names or pathogens are listed below each host species (host species in bold). Commonly used names are included.

This technique can also be applied on single nuclei (snRNAseq) instead of whole cells. The main downside of this being the loss of cytoplasmic RNA^503^ which results in lower numbers of genes per cell sequenced, and possible subsequent loss of well‐known marker genes. However, snRNAseq has the advantage of allowing samples to be flash frozen for storage, transportation, nuclei extraction and sequencing at a later date. Unlike whole cells, nuclei dissociation can be performed on ice, minimising heat stress and the subsequent loss of specific cell types.^160^, ^161^, ^504^ Robust nuclei dissociation protocols have been developed and shown to work well in different tissue types and produce results concordant with the sequencing of whole cells.^505^, ^506^

Single‐cell/nuclei RNA sequencing can identify changes in cell type, abundance, and transcripts between samples. By identifying transcriptomic signatures at the cellular level these technologies should provide a much more precise molecular understanding of disease resistance in aquaculture species than has been previously possible. For instance, single‐cell RNA sequencing has been used to characterise the immune system of the shrimp *Marsupenaeus japonicus*, identifying six different types of haemocytes with differentiated immune roles.^507^ Only a handful of other single‐cell studies have been carried out in aquaculture species, for example, on the immunologic profile of Atlantic salmon gill,^508^ leukocyte populations in *Nile tilapia* ^509^ and cold tolerance signatures in the hepatopancreas of whiteleg shrimp.^510^ Single‐cell technologies therefore have great potential for investigating the biological mechanisms underlying the phenotypic differences in disease response between species.

#### 4.1.3 Spatial transcriptomics

Spatial transcriptomics is an overarching term for methodologies assigning mRNA to their relative position in a tissue section (Table 1,^162^, ^163^). These technologies are primarily categorised as either imaging‐based approaches (in situ sequencing and in situ hybridization‐based methods), or next‐generation sequencing (NGS)‐based methods. While imaging‐based spatial transcriptomic techniques are typically limited to detecting a handful of genes that have been selected a priori, NGS methods indiscriminately target RNA within a spatial area which are mapped back to their spatial position using unique barcodes.^511^, ^512^, ^513^, ^514^, ^515^ NGS methods are therefore an attractive approach for explorative research, allowing for the spatial depiction of all gene activity in a tissue sample. In terms of ectoparasites such as sea lice, NGS methods could be used to compare fine‐scale localised gene expression at the site of parasite attachment. Applying the technique on vertical skin sections through the attachment site of the parasite may capture different responses between resistant and susceptible animals throughout the skin layers. For instance, immune cell infiltration is a well‐characterised feature of coho salmon resistance to sea lice,^72^, ^104^, ^516^ but we do not know what signals drive this response in coho, and why this response is weaker in susceptible salmon species. Spatial transcriptomics could be used to discriminate what signals are released by host cells at the attachment site at critical times post‐attachment, some of which might be key signals driving immune cell infiltration. Given that the spatial resolution of NGS methods is often greater than the diameter of a single cell, integrating spatial transcriptomic data with snRNAseq/scRNAseq data could facilitate powerful insights into transcriptomic differences by cell type through space.^515^ Tissue and cell level spatial proteomics is also possible^517^ and could be compared with spatial transcriptomic data to investigate connections between the transcriptome and proteome with respect to disease resistance.

#### 4.1.4 Proteomics

The study of infectious diseases requires defining the cellular and molecular mechanisms at the site of pathogen entry or attachment to a host. The interacting proteins of both species are likely to have a crucial role in the early infection process. Proteomics can decipher host‐pathogen cross‐talk, providing vital information on infection, early pathogenesis, host‐immune response and mechanisms by which pathogens evade host defence. Use of proteomics in aquatic species disease research is outlined in a review by Moreira et al.^518^ Recent years have seen major advancements in the technology, although most new developments have so far been applied to unravelling infectious disease mechanisms and pathogenesis in non‐aquaculture species, such as humans. Here, we highlight some of the advanced proteomic tools that will likely have application for investigating infectious disease biology in fish and shellfish.

Proteomics provides a vast array of tools, not only to measure the abundance levels of thousands of individual proteins across various states/conditions from nanogram levels of samples, but also for addressing challenges at proteoform and post‐translational modification levels. Global quantitative profiling employing stable isotopic labelling (e.g.,^519^) and extensive fractionation of peptides prior to mass spectrometry (MS) by various chromatography techniques give deep proteome coverage.

New generation high‐resolution MS systems combining additional gas‐phase separation of peptide ions based on ion‐mobility and therefore label‐free quantification approaches by either data‐dependant or data‐independent acquisition methods^520^ can be used to quantify proteins over a larger dynamic range from complex mixtures. Targeted MS methods can be used to study a set of proteins of interest in complex mixtures with high sensitivity, selectivity and quantitative reproducibility (e.g., with parallel‐reaction monitoring^521^).

Post‐translational modifications (PTM) often play a critical role in the biology of infection. It has been widely believed that only eukaryotes were able to make PTMs, however, now high‐resolution mass spectrometry has identified a plethora of PTMs in prokaryotic pathogens.^522^ While host cells can utilise a cascade of PTM changes to accelerate immune response, infecting virus also use PTMs to breakthrough host defence.^523^ UNIMOD, a database for PTMs detected by mass spectrometry has now reported over 1500 modifications, indicating the diversity and richness of post‐translational regulation, although the biological functions of many of these modifications are unknown (https://www.unimod.org/modifications_list.php, 30 May 2022).

Interactions between host and pathogen proteins are critical for pathogen replication, and for escape from and control of the host immune response. High‐resolution LC–MS‐based proteomics (e.g., using co‐immunoprecipitation with target‐specific antibodies conjugated to protein A/G affinity beads followed by MS‐based identification,^524^ proximity‐dependent biotin identification^525^, ^526^ with GFP binding nanobodies,^527^ cofractionation MS,^528^ clear‐native PAGE^529^ and/or cross‐linking mass spectrometry^530^) generate detailed quantitative protein profiles that could be used to reveal potential immunomodulatory host–parasite interactions.

In *P*. *vannamei*, GST‐pull down and mass spectrometry analysis were used to investigate the interaction of the immediate‐early WSSV protein (IE‐1) with shrimp proteins, finding 361 host proteins that could potentially bind to IE.^531^ Most of these proteins were involved in signalling pathways such as the prophenoloxidase (proPO), PI3K‐AKT, MAPK, focal adhesion, and cell cycle systems. Knockdown of IE‐1 reduced viral load and WSSV gene expression, while recombinant IE‐1 inhibited host prophenoloxidase in a dose‐dependent manner.^531^ Metabolomic studies of gill, haemolymph and hepatopancreas have revealed clearly different profiles in WSSV infected and non‐infected shrimp, consistent with changes in osmoregulation in the gills, upregulation of the glutathione pathway, increased production of an antimicrobial peptide itaconic acid in the hemolymph and a shift from aerobic to anaerobic metabolism as previously described.^532^ Interestingly, some of the changes in the haemolymph (increased TCA intermediates and decreased amino acids) were similar to those in haemolymph of *P*. *vannamei* challenged with *Vibrio parahaemolyticus* ^533^ while some molecules were specific for each pathogen such as itaconic acid in WSSV‐challenged shrimp and increased phosphoenolpyruvic acid (PEP) in shrimp exposed to *V*. *parahaemolyticus*, suggesting that there are pathogen‐specific innate immune responses in shrimp. The use of host‐targeted drugs that act on these pathways hijacked by viruses is now emerging as a potential source of antiviral treatments for human medicine.^534^

### 4.2 Semiochemicals

#### 4.2.1 Detection and characterisation

In marine ecosystems, obligate ectoparasites, such as sea lice, use chemical cues and other sensory signals to increase the probability of encountering a host and to identify appropriate hosts on which they depend to complete their life cycle. The chemical compounds that underlie host identification by the sea lice are not fully described or characterised. Identification of semiochemicals attracting parasites opens new possibilities for parasite control. Quantitative assays of parasite attractants (kairomones) or repellents (allomones) could guide the selection of favourable variants for breeding programs. The effect of rearing conditions and feeds on attractiveness can be mechanistically assessed. Moreover, semiochemicals can be used in push‐pull strategies^141^ using anti‐parasite fouling devices, ointments and baited traps.^535^ Traditionally semiochemicals are identified by bioassay‐guided fractionation. Crude extracts are separated into finer fractions based on chemical properties, for example, polarity, size, or volatility. Active fractions (proven by bioassay) are further separated into new fractions and the procedure is repeated until an active compound has been isolated. High‐resolution analytical methods are often now used in combination with statistical methods (metabolomics) to identify and directly bioassay candidate compounds.^535^, ^536^ The metabolomic approach can be used to identify candidate compounds involved in host–parasite interactions, and to distinguish those produced exclusively, or at markedly higher levels, by susceptible species or individuals. The effect of candidate compounds on the behaviour of parasites at infectious stages of development can then be assayed. To accentuate differences and improve the power of detection, samples from host individuals with extremely high and low levels of resistance to the parasite (based on estimated breeding values and/or direct counts post‐infection) could be tested. Non‐target species that are not parasitized can be used as negative controls. Candidate compounds may be water soluble metabolites released from the fish directly into seawater^145^ or compounds secreted in the mucous or released as faeces or urine. A full spectrum of semiochemicals could be obtained from water bathing the host using solid phase extraction.^145^ Sub‐nanogram quantities of the semiochemicals in the collected samples can be measured using a gas chromatography‐flame ionisation detector or ‐mass spectrometer multiple‐point external method with authentic standards of identified compounds.

Replicate filming tanks or arenas populated with copepodids have been used for rapid screening of behavioural responses (swimming speed, hop frequency, and rate of change of direction in response) to measure lice attraction/repulsion to compounds.^537^ The responses of lice to compounds in the behavioural tests are corroborated by neurophysiology experiments.^139^ Copepodids are stimulated to swim rapidly upward using a standardised light flash (generating a change of light intensity simulating a group of fish swimming overhead). Compounds of interest are added to the water at higher than natural concentrations to determine if they enhance the response to the standardised light stimulus. The behavioural data is digitally recorded and evaluated using motion analysis equipment that has been specifically developed for measuring such lice behavioural differences.^143^, ^146^, ^537^, ^538^ Many different compounds can be processed relatively quickly using this assay. Different concentrations of the validated lice‐attractant compounds can be used to generate stimulus–response curves. These stimulus–response curves serve as a basis to estimate the distance from a point source of salmon at which the compounds would activate free‐swimming copepodids.

Another line of research involves testing if exposure to semiochemicals provokes differential gene expression in copepodids (e.g., induces the production of immunomodulatory compounds). Salmon conditioned water and putative semiochemicals could be tested on batches of infective copepodid larvae. Exposed copepodids could be sampled at specific time points post‐exposure and their transcriptomic profile analysed using Illumina bulk RNASeq. Profiling of treated and untreated lice would inform as to whether the levels of louse host‐interacting proteins identified are also influenced by semiochemicals released by resistant or susceptible salmonid hosts.

To associate semiochemicals affecting host–lice interactions with candidate genes affecting semiochemical production, a focus could be on genes coding for enzymes involved in pathways that produce secondary metabolites. These may be absent/present in susceptible salmon species, differentially regulated or absent/present in resistant salmon species and absent/present in other marine species. In common with all lipophilic compounds, the metabolism of semiochemicals most likely involves cytochrome P450 (CYP) monooxygenases. The CYP superfamily is characterised by extremely rapid evolution and diversification, and frequent acquisition and loss of genes.^539^, ^540^ Some CYPs (e.g., the entire CYP2M1 subfamily) are found only in salmonid fish.^541^ Tissue profiles combined with gene expression data from existing transcriptome databases (e.g.^542^) and qPCR could be used to identify target genes for blocking the production of parasite attractants.

### 4.3 Gene editing to identify functional disease resistance genes

#### 4.3.1 Targeted editing of candidate loci in embryos and cells

The CRISPR‐Cas system^85^ is a powerful and versatile genome editing tool that can be used to knock out, knock in or to modify transcriptional regulation of target genes. Knock out of targets can be achieved by delivering a Cas enzyme such as Cas9 or Cas12a together with a guide RNA (gRNA) which guides the Cas enzymes to bind the target region and make double‐strand breaks (DSBs), which are typically repaired via non‐homologous end joining resulting in random insertion or deletion mutations (indels) around the DSB sites.^543^ Knock in, or other forms of precise editing, can be achieved by adding template DNA of the desired sequence containing homology arms together with the Cas and gRNAs, such that the DSBs are followed by homology‐directed repair (HDR).^543^ The transcriptional regulation of targets can be achieved by using modified dead Cas9 (dCas9) with transcriptional activators or suppressors for upregulation or suppression of the expression of targets.^544^ Finding an efficient method for the delivery of editing constructs into cells or embryos is critical for successful gene editing. There are three general pathways by which the components for gene editing can be delivered: (1) physical administration by microinjection, electroporation or hydrodynamics, (2) viral vector delivery or, (3) non‐viral vector delivery using, for example, liposomes.^545^ In addition, editing efficiency varies depending on gRNA sequence and is also influenced by features associated with the target sequence such as chromatin state which affects accessibility to the target region.^546^ So far there have been few applications of gene editing to species in aquaculture (Table 4). CRISPR‐Cas9 has been successfully used for both the knock out and in of genes in Atlantic salmon in vivo by microinjection into the zygote, and development of edited animals is becoming relatively routine^556^, ^557^, ^558^, ^559^, ^560^ as are knockouts in salmonid cell lines using electroporation of Cas ribonucleoprotein or using lentiviral delivery.^561^, ^562^ In contrast, attempts to perform CRISPR‐Cas9 genome editing in *L*. *vannamei* shrimp zygotes were unsuccessful using electroporation and chemical transfection.^563^ Further research on delivery methods of CRISPR‐Cas is required to achieve efficient in vivo genome editing in penaeid shrimp, for example via microinjection of CRISPR‐Cas9, as has been successfully applied to the decapod (non‐penaeid) shrimp *Exopalaemon carinicuda*.^555^, ^563^, ^564^, ^565^, ^566^, ^567^, ^568^

**TABLE 4:** Application of gene editing for boosting host resistance for species in aquaculture for food production. Commonly used names are included

Host species	Pathogens	Target editing
Atlantic salmon	Infectious pancreatic necrosis virus^154^	Knockout of NEDD‐8 activating enzyme 1 (*nae1*) and epithelial cadherin (*cdh1*)
Asian seabass	Nervous necrosis virus^378^	Knockout of ribonucleoside‐diphosphate M1 *(rrm1)*
Channel catfish	*Edwardsiella ictaluri* ^547^, ^548^ *Flavobacterium columnare* ^547^, ^548^	Transgenesis of *cecropin,* knockout of toll/interleukin 1 receptor domain‐containing adapter molecule (*ticam1*) and rhamnose binding lectin (*rbl*)
	Bacterial (e.g., *Acinetobacter baumannii* and *Klebsiella pneumonia*)^549^	Knock‐in of alligator cathelicidin
Grass carp	Grass carp reovirus^550^, ^551^	Knockout of junctional adhesion molecule‐A (gcJAM‐A)
	*Aeromonas hydrophila* ^552^	Transgenesis of human lactoferrin (hLF)
Rohu	Viral, bacterial, lice^553^	Knockout of toll‐like receptor 22 (*trl22*)
Rainbow Trout	*Aeromonas salmonicida* and infectious haematopoietic necrosis virus^554^	Transgenesis of cecropin P1 and synthetic cecropin B analogue (CF‐17)
	*Aeromonas salmonicida*, Infectious Haematopoietic Necrosis Virus and *Ceratomyxa shasta* ^409^	Transgenesis of cecropin P1
Ridgetail white prawn	*Vibrio parahaemolyticus* and *Aeromonas hydrophila* ^555^	Knockout of chitinase

Based on results from large‐scale and integrated genomic analyses, target genes could be chosen for the investigation of their functional role using genome editing tools. These targets could be candidate genes or pathways purported to underlie intra‐ or inter‐specific genetic variation in host resistance to the pathogen, for example, candidate genes within a QTL region. The targets may also be identified from knowledge of the biology of the host–pathogen interaction, for example, as was the case that led to the knockout of a domain of a cellular receptor causing complete resistance to the viral disease porcine reproductive and respiratory syndrome in pigs.^569^ Target loci for host resistance to sea lice might be those (i) upstream of immune pathways involved in successful lice rejection, (ii) putative targets of louse immunomodulation, or (iii) enzyme(s) required for the production of semiochemical(s) that are found to differ (in occurrence or gene expression) between salmon species with differing louse attachment or activity. Similarly, targets for WSSV host resistance would include (i) genes involved in the adhesion and entrance of WSSV into host cells such as the newly cloned chondroitin proteoglycan 2 of *Litopenaeus vannamei* (*LvCPG2*), which interacts with both VP26 and VP28 of WSSV facilitating WSSV adhesion and penetration into shrimp hemocytes,^570^ (ii) the family of NAD+‐dependent protein deacetylases sirtuins, that can regulate viral replication in vertebrates (in *P*. *vannamei* the silencing of LvSIRT1 was associated with a decreased gene expression in WSSV^571^) or, (iii) genes involved in activation of Toll, IMD signalling and JAK/STAT pathways, (iv) other genes affecting the up‐regulation or effectiveness of AMPs, (v) enhancers of NF‐kB signalling and, (vi) inhibitors of the PI3K‐Akt‐ mTOR pathway. Gene knock out using CRISPR‐Cas9 targeting early exons, or gene upregulation via dCas9 with a transcriptional activator, could be used as appropriate.

High throughput genome‐wide CRISPR screens may also be an effective route to use for identifying targets (Table 1). CRISPR‐Cas mediated genome‐wide gene functional screenings such as genome‐wide CRISPR knockout (GeCKO), CRISPR activation (CRISPRa) or interference (CRISPRi) screenings could also be used to identify genes involved in disease resistance.^572^ To perform such screenings, engineered cell lines expressing appropriate Cas effectors, and a valid way to deliver gRNA libraries into the cells, are prerequisites. To date, lentiviral delivery has been optimised in salmonid cell lines^561^ but genome‐wide CRISPR screenings have not yet been reported in aquaculture species.

#### 4.3.2 Testing the effects of edits on host resistance

Genome‐edited hosts, along with unedited and mock‐edited control animals of the same families can be challenged with the pathogen of interest to assess whether the gene(s) subjected to editing affect host resistance by measuring and comparing host survival and/or pathogen load, as has been done to explore the effect of the chitinase gene on ridge back white shrimp host resistance to *Aeromonas hydrophila* and *Vibrio parahaemolyticus*.^555^ DNA and RNA samples would be collected to check if the edit is present in the target organ and if it has the desired effect on gene expression. Tests for mosaicism (using PCR and sequencing) and checks for obvious effects on the external phenotype and behaviour should also be carried out. Detected signs of developmental abnormalities or high embryonic mortality rates will also inform whether particular edits should be pursued further. A full evaluation of whether there are side effects on other important traits (e.g., off‐target effects caused by unintended editing at other sites in the genome or ill‐effects resulting from disruption of the target gene) would require growing edited animals until harvest size.

## 5 TECHNOLOGIES AND APPROACHES FOR BOOSTING HOST RESISTANCE

### 5.1 Genomic selection

Genomic selection,^83^ which has been described by Goddard and Hayes^573^ as a form of marker‐assisted selection in which genetic markers covering the whole genome are used so that all QTL are in linkage disequilibrium with at least one marker, is now routinely applied to some aquaculture breeding programmes (Table 5).

**TABLE 5:** Application of genomic prediction and/or genomic selection for boosting host resistance for species in aquaculture for food production

**Atlantic salmon**	**Rainbow trout**
Amoebic gill disease^186^, ^187^	Bacterial cold‐water disease^574^, ^575^
Sea lice^66^, ^576^	*Piscirickettsia salmonis* ^577^
***Coho salmon***	Infectious pancreatic necrosis^578^
*Piscirickettsia salmonis* ^193^	Haematopoietic necrosis virus^579^
**European sea bass**	**Red tilapia**
Nervous necrosis virus^214^, ^580^, ^581^	*Streptococcus agalactiae* ^212^
*Vibrio anguillarum* ^582^	***Striped catfish***
*Vibrio harveyi* ^580^	*Edwardsiella ictaluri* ^583^
**Gilthead sea bream**	**Tiger pufferfish**
Nervous necrosis virus^580^	Heterobothriosis^584^
Phytobacteriosis^230^	***Whiteleg shrimp***
Pasteurellosis^581^	White spot syndrome virus^585^
*Sparicotyle chrysophrii* ^231^	***Pacific oyster***
***Japanese flounder***	Ostreid herpesvirus^586^
Edwardsiellosis^587^	

*Note*: Disease names or pathogens are listed below each host species (host species in bold). Commonly used names are included.

For traits measured on sibs or other relatives of the breeding candidates, that is, not on candidates themselves, genomic selection is expected to outperform traditional selection methods because it enables selection within families (resulting in better utilisation of genetic variance and a higher intensity of selection) and is more accurate for estimating breeding values. This is especially important for aquaculture species with a large number of fullsib candidates per family^588^ and increases the rate of genetic gain that is possible for a given rate of inbreeding. To use genomic selection instead of traditional pedigree‐based breeding value estimation has been shown to increase accuracy in aquaculture breeding schemes.^589^ Genotyping costs for genomic selection can be expensive due to the genotyping of the many individuals in the reference population (to achieve high accuracy), and large numbers of candidates (to achieve a high selection intensity). Therefore, approaches to reduce genotyping costs without compromising prediction accuracy are needed. Designs that aim to reduce genotyping by pooling extreme groups of individuals, such as those in which sibling DNA samples are pooled according to challenge test results, and those utilising genotype imputation, have been found to be effective.^590^, ^591^ Finally, the effects of different marker densities have been tested and optimised (e.g.,^592^). Estimation of breeding values for the selected candidates are based on the SNP effects estimated in siblings using GBLUP,^593^ various Bayesian methods or SNP BLUP.^83^ Single‐step genomic BLUP methods that combine information from genotyped and non‐genotyped individuals are also used.^594^ The integration of QTL information with genomic selection, and possible implications on the accuracy of predicted breeding values is a scenario under investigation.^185^

Choice of genotyping method can also be used to maximise the cost–benefit ratio. SNP chips are often used. These have some development costs, but give high SNP density, repeatability and reliability. For small populations, where the investment to develop a SNP chip may be high compared to the economic benefits of genomic selection, lower‐cost genotyping methods using lower numbers of SNPs have been shown in simulations to give high accuracy of EBV estimation when combining SNP and pedigree information^588^ or when using imputation to higher density SNP panels^595^ (depending on that some animals in the population have been genotyped for higher density). Genotyping‐by‐sequencing approaches such as RADseq can be used for species where no reference genome exists and/or if SNPs have not previously been detected.^596^ The genotyping needed for genomic selection may also provide practical benefits since young animals can be pooled at an early stage and relationships can be inferred later after genotyping, reducing the need for keeping families separate and for physical tagging.

Genomic selection has provided a powerful and accurate means of achieving high rates of genetic gain for WSSV resistance in *L. vannamei* shrimp.^585^ To run a traditional sib‐selection for WSSV resistance, families would have to be kept separate and then tagged before running the challenge test. Keeping families separate would introduce a common environmental effect of family confounded with genetics and is practically challenging requiring more facilities, space and physical tagging of young shrimp before the WSSV test could be performed. In this case, genomic selection not only increases accuracy and genetic gain, but it opens a possibility to select for traits that would not be feasible to include in a traditional breeding programme. For the case study of WSSV resistance in *L*. *vannamei*,^585^ one generation of genomic selection increased WSSV survival by 13 percentage units.

### 5.2 Gene editing

Application of gene editing in aquaculture breeding requires comprehensive consideration and discussion from technical, regulatory and public acceptance standpoints. Therefore, identification of a strong candidate to edit can be considered as just the first step towards application. For disease resistance traits, various genomic techniques can be applied to identify strong candidate genes, and their function can be investigated in vitro and in vivo using CRISPR‐Cas genome editing systems (as described above). If a particularly promising candidate edit is identified, gene editing may then also be applicable as a tool for the incorporation of favourable edits into aquaculture stocks, potentially resulting in a large genetic gain.^597^ Target edits could include de novo alleles identified in closely related species and need not be limited to naturally occurring polymorphisms segregating in commercial populations (e.g., causative mutations for major QTLs).^597^, ^598^ For example, genome editing could potentially enable transfer of the mechanisms of host resistance to sea lice from coho to Atlantic salmon, via modification of specific genes and pathways in Atlantic salmon to mimic the resistance mechanisms found in coho.^598^ The use of gene editing to create de novo alleles could potentially improve the resistance of populations beyond what could be achieved in a short time horizon via selective breeding alone.

For species with relatively long generation intervals (such as Atlantic salmon, 3–4 years), genome editing could be particularly beneficial for speeding up the development of disease resistance. However, because there are normally many traits of importance to aquaculture, and because inbreeding and loss of genetic diversity can reduce the fitness of populations, and both therefore need to be carefully limited, the propagation and dissemination of genome‐edited fish will almost certainly need to take place as part of a well‐managed selective breeding programme (rather than replacing such a programme). As such, the practicalities of how to incorporate editing technologies into modern aquaculture breeding programmes require careful thought and study. For example, mosaicism remains a major issue with the direct editing of animals via microinjection, and therefore obtaining germplasm fixed for a single desirable edit is both challenging and time consuming. Furthermore, it is conceivable that genome‐edited animals may be required to be sterilised to avoid any risk of interbreeding with wild conspecifics. Sterility can also be achieved via genome editing, but it is a particularly challenging trait to include into a breeding programme for obvious reasons, unless it can be easily reversed when reproduction is required. The use of germ cell technologies and surrogate broodstock may offer some potential solutions, via culture and editing of germ cells in vitro followed by gamete production from surrogate hosts.^599^ This approach could also disentangle the production and dissemination of edited germplasm from the breeding nucleus germplasm, such that sterility could be introduced, and edits could be tailored according to the producers' requirements. Finally, a major challenge after finding targets for editing is to determine whether the editing of such target genes otherwise impacts the biology of the animal. As an initial screen for off‐target effects, CRISRP‐Cas9 mediated gene functional analysis could be performed first in cell lines.^562^ In any case, further testing and optimisation of methods for accurate and efficient implementation of genome editing for aquaculture will be required before edited fish or shellfish can be widely disseminated onto farms and before any edited animals can be accepted and approved for human consumption.

### 5.3 Semiochemicals

#### 5.3.1 Feasibility as a phenotype for selective breeding

If semiochemical profiles are sufficiently correlated with lice density, then these profiles may have potential as a convenient phenotype for assessing lice susceptibility on breeding candidates and their sibs without challenge testing using lice. Targeted assay(s) for chromatographic analysis of semiochemicals from mucus samples could be developed for compounds that elicit proven behavioural responses in lice. Use of such assays might potentially enable avoidance of the need for costly challenge tests and large field trials. Tandem mass spectrometry using LC‐QQQ‐MS provides a suitable instrumental platform for this type of measurement. Intraclass correlation coefficients (i.e., the repeatability) of the repeated semiochemical profiles prior to the lice infection could be used as a measure of the temporal consistency of the semiochemical profiles released by the fish over time. The magnitude of the genetic correlation of lice density with the pre‐infection semiochemical profiles, and heritability estimates for each semiochemical, will determine whether some of these semiochemicals may be suitable phenotypic measures for indirect selection for increased lice resistance, or for use in other lice‐combatting strategies.

#### 5.3.2 Application of synthesised or extracted semiochemicals

Cost‐effective semiochemical production opens up new methods of reducing host–parasite encounters. Some existing commercial feeds are claimed to contain repellent semiochemical additives and European patents exist for some methods and feed compositions for masking fish semiochemicals (e.g., European Patent Number EP2517568A1). Research has shown that ectoparasite infections on fish (amberjack, Nile tilapia and rainbow trout) are reduced when the fish are grown using diets containing plant derived extracts or beta‐glucan additives^600^, ^601^, ^602^, ^603^ and proof‐of‐concept evidence has shown that botanically‐derived materials can be incorporated into feed additives to reduce the attraction of *L. salmonis* to salmonid host cues.^604^ Increased knowledge about genetic differences in semiochemical production between resistant and susceptible hosts to sea lice infection may help in the design of the formulation of feed additives for use to boost Atlantic salmon's ability to resist sea lice infection.

Several strategies could be used to design feed additives with potential to boost host resistance to sea lice. For example, ingredients that block odour receptors in the parasite, mask kairomones released from the host, and/or increase allomone production in the host could be tested. As a first pass test, ex vivo fin assays could be used to assess the anti‐attachment properties of a candidate dietary ingredient.^605^ Briefly, infectious copepodids are incubated with a candidate ingredient added to the water at different doses together with excised pectoral fins of Atlantic salmon. Copepodids readily attach to submerged fins under these conditions while the test ingredient may reduce the attachment rate if it possesses anti‐lice properties. The copepodids' phenotype (number successfully attached to submerged ex‐vivo fins, free swimming and moribund animals), as well as copepodid gene expression, could be used to assess the impact of several additives in a mid‐throughput manner and help in the prioritisation of candidate ingredients for further lice challenge trials. A more time and resource‐demanding variant of the ex vivo fin assay involves a short feeding in vivo trial that precedes the ex vivo fin assay. Atlantic salmon are exposed to the feed that contains the ingredient with suspected anti‐attachment properties, or the control feed, for 7–14 days prior to the ex vivo experiment to allow for the bioactive component(s) contained in the anti‐lice ingredient to accumulate in skin, including fins, where it acts as a repellent. Pectoral fins from the anti‐attachment feed group and control dietary group are removed from fish and placed in pairs (test and control) in glass containers filled with sea water and incubated with *L*. *salmonis* copepodids. Lower number of lice settling on fins originating from fish exposed to the test feed suggests that the tested dietary ingredient changes olfactory (or nutritional) properties of skin and mucus thus negatively affecting the early phases of lice attachment.^606^ Fin assays allow investigations of early processes that occur in copepodids around the time of attachment in a time‐series with many multiple time points.

Full trials to test the effect of feed additives are typically around 3‐month long, as they involve host acclimation, pre‐feeding of test diets (depending on the rate of accumulation of bioactives in skin and mucus, this period may need to last for up to a month) and an infection challenge with *L*. *salmonis* copepodids. Different dietary doses in triplicate tanks would need to be tested against the control dietary group. Growth rate and other important traits would be monitored throughout the trial to allow for the detection and assessment of any possible negative effects caused by the tested ingredient. All treated fish would be challenged with lice and resulting infestation densities recorded and compared to control groups to allow for the utility of feed additives for repelling lice and masking salmon to be assessed.

An alternative to boosting host resistance through anti‐attachment feeds is to deploy semiochemicals beyond the host to reduce the number of lice entering sea cages. This might be achieved using baiting traps around the perimeter of the farm, slow‐releasing decoy semiochemicals at a distance from the farm, or slow‐releasing repellent/masking semiochemicals within the farm footprint to discourage lice. Behavioural responses to host/non‐host cues are well‐documented in the laboratory,^136^, ^140^ yet the best evidence for efficacy in the sea was produced by a trial of cages with semiochemical‐impregnated mesh (^607^ reviewed in Reference [46]). Applications of semiochemicals outside of the host may not work at commercial scale, simply because the swimming capacity of louse larvae, which is sufficient to intercept a host over very small distances (millimetres to centimetres, as discussed in Reference [141]), may not be sufficient for most larvae to reach discrete trapping points or avoid an object the size of a commercial sea cage. Impregnation of cages with semiochemicals that either disrupt host‐finding once larvae enter the cage (e.g., by blocking semiochemical receptors while lice are within the semiochemical plume) or attract lice to the mesh itself (including a method of trapping or killing larvae that are attracted to the mesh), are approaches that could be tested.

### 5.4 Vaccines

Fish vaccination, which has been in use for over 40 years, has significantly contributed to the sustainability of the industry, and dramatically reduced the use of antibiotics.^10^ Most licensed vaccines currently used in aquaculture are produced using conventional methods and consist of inactivated or live‐attenuated whole organisms, predominantly whole cell bacterins. In aquaculture, commercially available vaccines using modern technologies, targeting specific pathogen components, only include recombinant or subunit protein vaccines.^10^ Examples include an *E*. *coli*‐based expression subunit vaccine against infectious pancreatic necrosis (IPN) in Norway (produced by Merck Animal Health) and a yeast‐based subunit vaccine against infectious salmon anaemia (ISA) virus available in Chile (manufactured by Virbac‐Centrovet).

Even though traditional vaccine technologies have enhanced livestock productivity, the efficacy of inactivated vaccines can be suboptimal and live‐attenuated ones may present safety concerns. To circumvent some of these flaws, veterinary medicine has been at the forefront of novel vaccine technology, pioneering the development and licensing of third‐generation vaccines including DNA, RNA and recombinant viral‐vector vaccines.^608^

The latest vaccine technology to be applied to aquaculture uses self‐replicating RNA vaccines based on an alphavirus genome (i.e., salmonid alphavirus 3).^609^ Alphaviruses contain RNA replication machinery which is left intact in the vaccine, and genes encoding for structural proteins that are replaced with the antigen of interest. Such antigen‐encoding RNA replicon platforms enable the production of a large amount of antigen from a small dose of vaccine. RNA vaccines have been found to give high protection against infectious salmon anaemia depending on the route of administration.^610^, ^611^

It is challenging to develop vaccines against parasites. In the case of ectoparasites, such as sea lice, there are even greater challenges because of the antigenic complexity of metazoan organisms and physical separation between the pathogen and the host, allowing the pathogen to conceal a large proportion of its potential antigens. Despite these difficulties, and despite the few published research initiatives in this area (Table 6), researchers have recently demonstrated the protective effect of using a salmon lice‐gut recombinant protein (P33) as a vaccine antigen against sea lice (*L*. *salmonis*) in a laboratory‐based trial.^613^ The identification of suitable antigens against sea lice followed a translational approach successfully implemented in the development of vaccines against ticks^614^, ^620^ by proteomic identification of louse feeding‐associated proteins. Under field conditions, a tick vaccine (Gavac; Heber Biotec), which is the only commercially available vaccine against an external parasite, uses a single tick gut antigen (Bm86) to induce protection against ticks (*Rhipicephalus microplus*) in cattle. The successful development of this tick vaccine, and the promising results observed in salmon immunised with the P33 vaccine have been achieved thanks to recent advances in ‘vaccinomics’, based on transcriptomic and proteomic data^620^ which allow a deeper understanding of the genetic factors and molecular pathways involved in the host–parasite interface. However, vaccines against lice may not be 100% effective, in which case they will not eliminate the lice problem but may reduce the number of lice per fish and/or the number of eggs produced per lice. This is the case with the tick vaccine for which 55%–100% efficacy in control was achieved 12–36 weeks after the first vaccination and 60% reduction in the number of acaricide treatments was achieved relative to that for non‐vaccinated cattle.^621^ If salmon lice vaccinations are not completely effective, animals may have to be revaccinated in the grow‐out phase, which is a demanding and costly procedure as the fish grow larger, and other supplementary measures (such as biological control, medicines and selective breeding) will still be needed.

**TABLE 6:** Published sea lice vaccines and antigen discovery platforms

Vaccine antigens	Antigen localisation/putative function	Antigen discovery technology	Administration method	Efficacy	References
Crude extract: Whole adult antigens	Gut antigen cocktail	Antigens characterised ConA enrichment and 1D SDS‐PAGE/immunoblotting	Injection (intraperitoneal)	*Lepeophtheirus salmonis* 26% reduction in gravid female egg numbers.	[612]
*Escherichia coli* expressed recombinant protein: p33—potassium cholride amino acid transporter	Gut: Cellular hypotonic salinity response and transmembrane transportation	RP‐LC–MS/MS proteomic identification of gut‐associated proteins	Injection (intraperitoneal) with IP boost	*L*. *salmonis* 41.3% reduction in chalimus. 35.7% reduction in adult lice.	[613, 614]
*E*. *coli* expressed recombinant protein: p30—putative toll‐like receptor 6	Gut: Innate and adaptive immune response	RP‐LC–MS/MS proteomic identification of gut‐associated proteins	Injection (intraperitoneal) with IP boost	*L*. *salmonis* 31% reduction in chalimus. 16% reduction in egg string length.	[614]
*E*. *coli* expressed recombinant protein: p0 ribosomal protein	Immunogenic midgut protein involved in assembly of 60S ribosomal subunit and protein synthesis	Sequencing data—low amino acid sequence similarity between louse and host (preventing tolerance or autoimmunity)	Injection (intraperitoneal) and immersion	*L*. *salmonis* 21% reduction of adult females, 42.5% reduction in gravid adult females. Delayed hatching of gravid female eggs, low copepodid counts in F1 generation.	[615, 616]
*E*. *coli* expressed recombinant protein: my32‐ls/akarin‐like	Transcription factors required for NF‐k‐dependent gene expression	Sequencing of conserved genes using degenerate primers and cloning	Injection (intraperitoneal)	*C*. *rogercresseyi* 57% reduction in adults.	[617, 618]
*E*. *coli* expressed recombinant protein: IPATH® Iron binding domain of *s*. *salar* transferrin and *S*. *salar* ferritin subunit H	Iron‐chelating – host blood iron regulatory proteins	Whole transcriptomic analysis/RNAseq using MiSeq Illumina^(R)^ sequencing	Injection (intraperitoneal)	*C*. *rogercresseyi* 78% reduction of adults vs. 10% in controls. Disrupted embryogenesis, genital segment inflammation. Significant reduction in adult lice burden (mean 17 control vs. 407 in vaccinates)	[619]

Transcriptomic profiling of differential responses to resistant and susceptible salmonid hosts has facilitated progress in understanding the importance of nutritional immunity in response to *Caligus rogercresseyi* infection and subsequent application of vaccination modulating iron‐chelating activity.^108^, ^619^ Injection of shrimp, *L*. *vannamei*, with recombinant ferritin has also demonstrated protective efficacy against WSSV by inhibiting viral replication.^622^ Another recombinant approach which is more feasible to apply at large scale includes double‐stranded RNA targeting VP28 (dsVP28) produced by the probiotic bacteria *Lactococcus lactis*. Administration of *L*. *lactis* expressing dsVP28 to the animals before the WSSV challenge, significantly increased survival and decreased viral load compared to non‐treated animals.^623^ Differential infection‐associated *L*. *salmonis* transciptomic profiles between Atlantic and Pacific salmonid hosts has also revealed potential virulence genes,^113^ which may be exploited for vaccination.

Sequencing of homologous or conserved genes based on proteins and vaccine targets of ticks and other arthropods has proven an effective approach in antigen mining for sea lice.^615^, ^616^, ^617^ RNAi enables functional characterisation of vaccine candidates^624^ and has been applied to characterise a number of potential sea lice vaccine candidates including biomolecules involved in egg production (e.g., yolk‐associated protein; LsYAP,^625^), digestion (e.g., KEDL receptor; LsKDELR and vesicular coatomer protein complex; LsCOPB2,^626^), muscle activity (e.g., LsalMS,^627^) and immunomodulation (e.g., prostaglandin E‐synthase 2; PGES2,^628^) leading to impaired parasite reproductive capacity, digestion and development. Gut digestion‐associated serine proteases, for example, trypsins have been characterised based on sequence homology with other crustacea,^629^ secretory products such as metallopeptidases and collagenases have been identified by proteomics using LC‐ESI‐MS/MS,^110^ and exocrine glands associated with attachment and host–parasite interactions have been mapped and characterised microscopically.^107^ Higher resolution sn‐RNA‐Seq and spatial‐ ‘omics’ technologies (Figure 1) will further elucidate the role glands and their secreted products play during louse infection, and these technologies are expected to result in the identification of new vaccine candidates.

## 6 OPTIMISING IMPLEMENTATION AND DISSEMINATION TO ACHIEVE POPULATION‐WIDE HOST RESISTANCE

The high cost of pathogen infection to the aquaculture industry is largely associated with the technology and labour involved with prevention, treatment and associated stresses, reduced growth and increased mortality thereafter (e.g., the high cost associated with delousing Atlantic salmon,^27^, ^36^, ^630^). For strategies boosting host resistance to be effective preventative measures, we need to ensure that they eliminate or severely reduce the need for treatments such as delousing across the industry and are everlasting or sustainable.

While we can focus on the genetic basis of host resistance, it is important to have one eye on the broader population dynamics at play. There is little value, for example, in producing a host salmon or shrimp with twice the resistance if this does not translate into a dramatic reduction in actual infestation rates throughout the entire production cycle. Such nonlinear returns are entirely possible as implementation scales up from an individual to population level, especially for instance if the modification introduced can be repaired (as has been a problem when CRISPR gene drives are introduced into wild populations, for example,^631^) or has little effect on the overall epidemiology of the disease. Alternatively, minor changes to resistance might have major consequences for infestation rates and may impose selection pressure on the parasite. Modelling of host–parasite dynamics, then, will provide an important tool for understanding how genetic manipulations might have maximum effect over a prolonged period. We focus here mainly on salmon louse as a case study. The ideas we discuss are, of course, relevant to the shrimp example also, but the shrimp example is more complex and critical factors (such as spatial connectivity) are not yet well resolved for WSSV.

### 6.1 Understanding the epidemiological effects of genomic solutions

The full scope of genomic solutions to control infectious diseases in aquaculture is likely to be grossly under‐realised because current selection does not consider host–parasite interactions and resulting epidemiological effects.^632^ This is particularly pertinent in aquaculture programmes, in which disease resistance is defined as the ability to survive when exposed to infectious pathogens, as is the case for most virus infections, including WSSV in whiteleg shrimp.^585^ Survivors may have higher tolerance to cope with infections and thus transmit infections to others.^79^ Few studies consider how endurance is affected by selection for disease resistance.^234^ Hence, it is generally not known whether and to what extent selecting directly on survival as the resistance phenotype reduces disease transmission and thus the incidence and severity of disease outbreaks in populations.^633^ Epidemiological models for micro‐parasitic infections, such as virus infections, point to three underlying epidemiological host traits that affect pathogen transmission and subsequent mortality rates: susceptibility (the propensity of an uninfected individual to become infected when exposed to infectious material), infectivity (the ability of an individual, once infected, to transmit the infection) and mortality (the propensity of an infected individual to die, i.e., the opposite of tolerance). Evidence from recent transmission experiments in aquaculture populations suggests that substantial host genetic variation and co‐variation may exist for all three host traits, and that all three epidemiological traits can be reliably estimated.^79^, ^634^ Furthermore, genetic‐epidemiological models indicate that genomic selection for reduced host susceptibility and infectivity may more effectively reduce disease prevalence than current selection for disease resistance.^635^

Genetic selection for host resistance to external‐parasites like sea lice is expected to directly reduce parasite load in the populations, as observed in practice for sea lice infections in salmon.^46^ This is because parasite count is used as the resistance phenotype. For example, resistant fish with lower sea lice counts are expected to also propagate fewer lice and hence reduce the overall lice prevalence in the population.^60^ Nevertheless, the full potential of genomics is also likely to be under‐realised for these types of infections. There are many host resistance mechanisms that interfere with the parasite life cycle that are likely under host genetic control and could therefore be targeted for genetic improvement. These include, for example, those that lead to (a) an increasing proportion of non‐gravid and thus non‐reproductive females due to a reduction in the average lice density per fish^636^; (b) reduced fertility of the gravid female lice; (c) lower hatchability of the eggs; (d) lower survival from hatching to the infective copepodid stage; (e) lower infective success rate of the copepodids; (f) lower survival success of copepods through behavioural divergence or lower quality energetic resources. Factors (b)–(f) may be caused by lice maturing on fish with increased resistance to lice thus leading to an inferior rearing environment due to, for example, different chemical factors in the mucus and skin of the salmon host. This is expected to result in a higher genetic gain in resistance to the lice than predicted from classical quantitative genetic theory. Research is needed to assess the host genetic effects underlying such mechanisms. In the first instance for the lice example, it would be useful to assess the differences in the number of male lice and the number of gravid and non‐gravid female lice, number of eggs per female lice, and infectivity of lice growing on fish with very high and low estimated breeding values for lice density.

A better understanding of the influence of host resistance on parasite epidemiology will allow the development of more efficient strategies to reduce lice infestation, including evaluation of the economic importance of performing genetic improvement for increased resistance of the host to the parasite and formulation of functional feeds. In particular, such data would inform epidemiological models of parasite prevalence in host populations that differ genetically in terms of their impact on the parasite life‐cycle.^60^ Such models are needed for optimising selective breeding strategies and furthering our understanding of the effect of selection for increased parasite resistance on possible correlated epidemiological effects through reduced parasite reproduction.

### 6.2 Mitigating possible counter‐evolution by the parasite or pathogen

It is well known that parasites and pathogens can rapidly adapt to pest controls used on farms.^637^, ^638^, ^639^, ^640^ Without proper precautions in place, pest evolution can lead to a dramatic decline in the efficacy of a novel control technology. The Atlantic population of *L*. *salmonis* has already evolved pesticide resistance to most of the chemical treatments used on salmon farms,^48^, ^641^ and there are concerns that they could similarly adapt to the various non‐chemical alternatives that are in use.^47^ Due to the strong louse gene flow between farms, advantageous traits can spread through the parasite population in a very short space of time.^641^, ^642^, ^643^ When developing new, parasite‐resistant strains of host, it is therefore imperative that the risk of counter‐adaptations evolving in the parasite is full assessed.

Just as individual Atlantic salmon vary in their ability to resist lice,^62^, ^188^ so too may there be genetic variation within *L*. *salmonis* in their ability to tolerate, evade or modulate the hosts' immune defences. Lice with improved infestation success and survival on resistant salmon strains would therefore be selected for. The possibility for interaction effects between salmon and louse genotypes on infestation success is yet to be studied.

In terrestrial agriculture, pathogens have evolved to overcome resistant genes in plants on many occasions.^644^, ^645^, ^646^, ^647^ Increased use of transgenic crops that have been modified to produce insecticidal proteins has driven a surge of counter‐adaptations in insect pests.^640^ However, selective breeding for resistance in animals has proven to be much more durable against counter‐adaptations, providing long‐standing protection against parasites.^648^, ^649^ Nevertheless, host–parasite co‐evolutionary arms races occur in many animal systems,^650^, ^651^, ^652^, ^653^ and so the potential for lice to adapt to changes in host resistance must be considered. Theoretical studies on parasite adaptation in hosts with different genetic resistance to the parasite suggest that it is unlikely that the parasite would change significantly as a result of selective breeding for increased parasite resistance.^654^ This is explained by the fact that adaptation of the pathogen requires allele(s) that are favourable in hosts with improved resistance, that long‐term selective breeding for increased disease resistance is a dynamic and broad spectra strategy involving very many genes and that the resilience in the host is rarely complete, varies between fish, and the genetic progress is relatively small per generation of breeding. This is why strategies involving selective breeding for host resistance to lice are probably less risky than, for example, drugs or vaccines that affect one or a few specific mechanisms of interaction between the host and the pathogen.^648^

*Lepeophtheirus salmonis* populations in the Atlantic and Pacific Oceans belong to two allopatric subspecies that likely diverged ~5 million years ago.^655^, ^656^ Given that parasite evolution is profoundly shaped by the host environment, one would expect the Atlantic and Pacific louse subspecies to have locally adapted to their sympatric *Salmo* and *Oncorhynchus* hosts, respectively, to some degree over these ~5 million years. This topic has received limited research but will be relevant if resistance mechanisms in Pacific salmon species are used as the template for gene‐edited Atlantic salmon. That Pacific species such as coho salmon have retained their relatively high resistance, despite millions of years of co‐evolution with lice, suggests that lice will be unlikely to overcome coho genes used as the basis for gene edits to produce resistant Atlantic salmon.^648^ Nevertheless, the full efficacy of this strategy could erode over time through local adaptations in the louse population. In the wild, coho salmon can still support relatively high louse infestations.^657^, ^658^, ^659^

Pacific salmon lice show an enhanced transcriptomic feeding response on Atlantic salmon compared with on Pacific hosts.^113^ Louse responses on sympatric coho and sockeye salmon were similar, despite these host species differing in resistance.^113^ Fast et al.^100^ measured the production of enzymes secreted by Pacific and Atlantic lice, thought to assist in digestion and/or host immunomodulation, in response to the mucus of different host species. Both louse subspecies had higher enzyme production when exposed to the mucus of Atlantic salmon, than when exposed to more resistant coho salmon (*O*. *kisutch*). Interestingly, mucus tended to stimulate a stronger feeding response in sympatric, rather than allopatric, louse subspecies.^100^ That is, Atlantic salmon mucus and coho mucus stimulated relatively higher enzyme secretion in Atlantic and Pacific lice, respectively. These differences in the feeding activity of lice on sympatric and allopatric host species^100^, ^113^ highlight the heightened susceptibility of Atlantic salmon to lice and are also suggestive of some degree of local adaptation.

Research into infestations on non‐salmonid hosts, such as the three‐spined stickleback (*Gasterosteus aculeatus*), may also provide insights into the extent to which lice can adapt to alternative host strains. *Lepeophtheirus salmonis* will readily infest sticklebacks in the laboratory and the wild, but do not appear to be able to successfully sexually mature on them.^660^, ^661^

Given the effort required to research and develop new mechanisms of host resistance, it is desirable for a strategy to maintain a high level of efficacy for as long as possible. A key plank of any plan for implementing genetic technologies for host resistance should be to minimise the risk of counter‐adaptation by the parasite. An evolutionarily stable strategy consistent with animal welfare and production demands is therefore needed. One approach for achieving this is to maintain genetic diversity in host resistance, both within and between host strains. The more genes for resistance that are incorporated into a host strain, the more complex the mechanism of resistance, and the harder it is for parasites to evolve counter‐adaptations.^662^, ^663^, ^664^ Of course, the trade‐off of this is the extra effort required to develop additional gene edits and focus selective breeding on disease resistance while maintaining high levels of genetic diversity in the captive‐bred populations.

Another approach for slowing the spread of parasite counter‐adaptation might be to establish refugia—in the salmon example, maintaining populations of susceptible salmon strains.^640^, ^665^, ^666^ Refugia are particularly effective at preventing parasite adaptation if counter‐adaptions incur fitness costs on susceptible host strains.^666^, ^667^ Wild populations can act as refugia, provided they are large enough.^665^, ^668^ In the Atlantic, however, farmed salmon are significantly more abundant than wild hosts.^34^ It might be more effective, then, if some farms were to act as refugia instead, by being stocked with susceptible host strains, but it seems unlikely that such deliberate degradation of fish welfare on a whole‐farm basis would be acceptable to the authorities and public.

Choosing which farms must forego resistant hosts to avoid counter‐adaptations would pose a difficult decision, as would decide how many different gene edits are needed to provide an evolutionarily durable strategy. This is where evolutionarily dynamic metapopulation models can be powerful tools (e.g.,^669^). Such models have previously been used to predict the rapid spread of pesticide resistance, and to identify evolutionary hotspots in areas of intensive farming.^669^ To simulate counter‐adaption to resistant hosts, these models could include the stocking of different host strains, selection imposed by these strains on louse genotypes, and recombination and mutation of louse genes. Model simulations can then be run for different scenarios to determine the optimal management regime: one that prevents lice from adapting to resistant hosts, while still significantly reducing lice infestations in the short term.

These models are valuable for identifying the most evolutionarily durable avenues for achieving host resistance. Once resistant strains have been developed, models can again be used, this time to inform how resistant salmon should be introduced to farms through space and time. Simulations can also be used to consider how resistant salmon should be deployed in concert with other preventative strategies,^46^ to further mitigate the risk of louse evolution.^670^ Of course, the accuracy of such models is directly proportional to our understanding of counter‐adaptations in lice. The more that is known about the mechanisms, genetic architecture and fitness costs behind any counter‐adaptations, the greater the predictive power of the model.

### 6.3 Integration of genomic technologies and dissemination to the sector with the implementation of gene editing and genomic selection

Genome editing in combination with other biotechnological advancements such as germ cell technologies could be used to help accelerate genetic improvement.^599^, ^671^ Gene editing can rapidly introduce favourable changes to the genome, either by fixing alleles at existing trait loci, creating de novo alleles or introducing alleles from other strains or species. However, seamless integration of genome editing technologies into a well‐managed breeding programme is required to ensure continuous genetic improvement and careful management of genetic diversity. It is also important to consider that gene editing is likely to alter the genetic architecture of other traits of interest, perhaps leading to substantial epistasis, and this would have to be accounted for in future breeding strategies.

The expected genetic improvement of traits of interest through selective breeding depends on several factors including heritability, selection intensity, selection accuracy and generation interval. The implementation of genome editing in aquaculture breeding will have the same goals as traditional selective breeding practices. In the cases here, the ultimate goals for genetic improvement should be to eliminate or reduce the number of delousing events required by the salmon industry and to boost the survival of shrimp in the face of WSSV disease. The number of lice that have infected the fish in challenge and/or field tests are the index traits that are being used for the salmon lice case whereas survival after a challenge test is the index trait currently used to select for WSSV resistance. However, host resistance is a complex phenotype consisting of multiple layers (attraction, prevention of attachment or infection, immune response, infectiousness, etc.). More detailed knowledge about host influences on disease reproductive success, host immunology and host semiochemicals influencing lice attraction and attachment could provide more specific phenotypes for host selection and enable more accurate forms of genomic selection for host resistance. Such knowledge will also provide targets for gene editing.

The effect of gene editing on disease resistance could be on a similar scale to that of a major gene affecting a trait, or in the case of a de novo edit, could be on a much larger scale (effecting disease resistance to an extent not seen within the breeding population). If the gene edits were directed at genetic variants causing QTL effects (in a way that creates similar variants to those that naturally exist in the population), the editing could have a potentially large impact on traits where one or a few major genes affect the trait considerably. In this case, the gene editing might bring us a large step forward by fixing a preferred allele for a gene underlying a QTL. For traits with a more polygenic structure, traditional selection and genomic selection would be preferred to this type of gene editing in most instances. In any case, the application of selection methods will improve the trait further, by selectively improving the genotypes at other loci related to the same trait.

However, as noted above, editing may alter the genetic architecture of the trait and lead to substantial epistasis. In plants, gene editing has been suggested as a final step after selection for complex traits, to improve monogenic quality traits.^672^ In aquaculture, gene editing needs to be done before selection for other traits has occurred. The edited fish might not therefore be chosen as a parent if they have poor performance for other traits of interest. Alternatively, surrogate broodstock technologies^599^ could help to reduce the long generation interval in Atlantic salmon and be used as a dissemination tool because the editing could be implemented after selection and before dissemination to producers without altering the breeding programme germplasm. The costs associated with these strategies (increased direct costs and/or reduced gain for other traits) may be acceptable if the gene editing is successful, important and does not need to be repeated every generation. However, if editing needs to be performed repeatedly, to cover many genes or traits, a more integrated approach is needed where for instance a few edited and selected individuals are used to introgress the edited variant into the population.

Editing efficiency also determines the feasibility of gene editing in aquaculture breeding. Studies on the optimal number of edited animals required in aquaculture breeding schemes are lacking, but simulation results from livestock schemes demonstrated that genome editing technologies in combination with genomic selection have the potential to increase genetic gain many‐fold for traits of interest compared with genomic selection alone.^673^, ^674^, ^675^ As of today, no cost–benefit analysis has been done for gene‐edited assisted selection (GEAS) programmes in aquaculture. Such an analysis would consider the number of gene‐edited individuals, single or multiple gene edit effects, other traits under selection, expected genetic gain and rate of inbreeding. Also, the rate of dissemination and the economic implications of these schemes should be assessed and compared with conventional breeding designs. Specific GEAS schemes with special attention to multiplier parents, which produce seed for the production tier, could then be designed. The multiplier parents carrying resistance edited genes to be selected, and the effect of this selection strategy on the resistance of the production population, should be investigated. A recent modelling study revealed that gene editing, when effectively combined with other disease control strategies, such as vaccination, may be able to eliminate persistent livestock diseases that are currently difficult to control.^676^ However, according to this study, such desirable outcomes can only be achieved if the distribution of genetically resistant individuals into commercial populations is coordinated by breeding companies or national schemes.

## 7 ETHICAL CONSIDERATIONS

Improving disease resistance improves the economics of aquaculture, and it also contributes to more sustainable aquaculture production by improving animal welfare and reducing the impact of aquaculture on wild stocks.^677^ Traditionally, selection for genetic improvement is based on survival information from challenge‐tested siblings of the breeding candidates, which involves exposing large numbers of individuals to pathogens, and therefore results in some suffering. Survival phenotypes are recorded from numerous relatives to enable accurate estimation of breeding values. This sacrifice can be argued to be ethically acceptable as it is done for avoiding suffering of a much greater number of individuals in large‐scale disease outbreaks. Even so, the intrinsic value and welfare of fish, independent of their utility to humans, should be part of our ethical considerations.^677^ Adoption of advanced breeding strategies (e.g., genomic selection) and/or adoption of new biotechnological methods (e.g., gene editing) will reduce the use of experimental animals. However, other ethical dilemmas arise when technologies such as gene editing are proposed to be adopted and implemented in practical breeding programmes.

Knowledge particular to each case is needed so that we can understand and evaluate potential off‐target and/or pleiotropic effects of gene editing on welfare. Another important consideration will be how the edits should be most effectively and responsibly created and spread throughout the breeding and multiplier populations. To ensure sustainable implementation of gene editing in aquaculture it is important to ensure that there is no possibility for edited fish to detrimentally affect wild population gene pools. The effect of escapees from a gene‐edited population into the wild is not only dependent on the number of escaped fish, but also their phenotypic characteristics related to fitness and the existing diversity in the receiving ecosystem.^678^ Genetic introgression from aquaculture to the wild populations could be avoided by making the gene‐edited fish sterile (either through editing of a locus affecting fertility or other means). In summary, the application of gene editing to aquaculture needs to be safe for the fish, the consumer and the environment^679^ and every proposed edit needs to be researched and evaluated with risk analyses on a case‐by‐case basis.

Research institutes producing gene‐edited fish and shellfish need to ensure future sustainable and beneficial impacts from the use of gene editing by developing strict guidelines for a *Responsible Research and Innovation* (RRI) framework. RRI frameworks commit to (1) describe, analyse and openly discuss the consequences of the process and outcomes of the research activities (anticipation), (2) holistically reflect on the moral, political and social assumptions and the main motivation and drivers of the research in question (reflexivity), (3) invite all relevant stakeholders to contribute views and opinions about the research trajectory (inclusion) and (4) be willing to change the trajectory of research if feedback from the stakeholders reflects that research is not likely to meet the requirement and expectations of the society (responsiveness).^680^ Rigorous quality systems need to be set in place to ensure that such guidelines are followed and that the fish and shellfish produced are securely contained.

Due to the complexity of the biotechnology, fish biology and of each specific situation, a case‐by‐case comprehensive and systematic approach is needed to address the ethical acceptability of each technological advance.^681^, ^682^ Ethical matrix (EM) approaches rely on generally accepted ethical principles and are used to get an overview of the most important ethical interests or values connected to the particular issue.^681^ The formulation of the overview involves and considers all groups who have an interest in or are affected by the technology including industry, NGO and government, and considers effects on the individual animals that are directly targeted, as well as the overall effect on nature. Emphasising the principles of well‐being, justice and fairness, the EM was designed to create discussion over ethical prioritisation for those with little or no training in ethical theory. EM can be used as a tool both to guide the research process within a project group and to create a dialogue between scientists and stakeholders. EM approaches can be used repeatedly and involve different stakeholder groups to promote the inclusiveness and responsiveness of the research project.

Any dissemination plan produced should follow and expand on institutional RRI guidelines that have developed for the use of gene editing (e.g.,^680^). Following the RRI dimension on inclusion, data should be shared and workshops held involving diverse scientific and industry representatives so that insight is gained, tapping into broad interdisciplinary knowledge and practical farming experiences. Furthermore, industry partners should be consulted at every step and be represented on the steering committee for such projects. Methodological ethical considerations include, for example, performing a priori power calculations to guarantee adequate but not unnecessarily large experimental setups, execution of challenge tests in bio‐secure environments to prevent pathogenic, parasitic, or edited material from escaping en masse into the surrounding environment, close monitoring of experimental animals and euthanasia of suffering individuals. In addition to using breeding strategies and technological methods that minimise challenge tests, more focus should be put towards assessment of alternative less invasive phenotypes (as an alternative to exposing fish to pathogens in challenge test).

With the work that is underway around the world applying genetic technologies to combat infectious diseases in aquaculture, aquaculture sectors will soon face important decisions. Likely there will be a much greater understanding of natural processes affecting host resistance to parasites and pathogens and strategies will be developed that could transform these industries as we know them. The projects now underway will advise on the most responsible and effective approach, or combination of approaches, for reducing or eliminating these parasite and disease problems. There will be a need for further testing of gene editing to ensure there are no risks. The aquaculture sectors involved need to have an open dialogue around the use of these technologies. ‘Business as usual’, with no change, is not a viable option, considering the effects of these diseases on profitability, animal welfare and public perceptions. The goal of such research will be to develop safe and effective strategies. But it is the aquaculture industry sectors involved that will need to work with relevant public and government agencies to decide if these strategies should be implemented, and to clear the path, so that they can solve this problem and transform their businesses.

## 8 CONCLUSIONS

Substantial research programmes are underway that aim to produce new knowledge that could be applied for boosting host resistance to eliminate or severely reduce infections by, for instance, sea lice in salmon and WSSV in shrimp. These projects are utilising a suite of technologies that have been enabled by ultra‐high‐throughput sequencing, such as single nuclei and spatial transcriptomics and SNP GWAS. Newly developed methodologies like in‐vivo or in‐vitro gene editing and functional testing hold great promise for helping to find and test genetic mechanisms affecting host resistance. These projects are also exploring the possibility of using genomic selection and gene editing with CRISPR‐Cas9 to create host populations that will resist these diseases. The implementation of these technologies needs to be carefully considered. Practical methods that will allow easy adoption, implementation and dissemination by aquaculture sectors are needed. Population genetic variability needs to be maintained, inbreeding limited and possibilities for the genetic improvement of other important traits must be ensured. Ethical concerns, particularly about the use of gene editing methodology, need to be openly discussed and debated in public arenas, and thorough testing and safeguards (e.g., sterilisation) are needed to ensure that there are no negative consequences for the wild populations of these species or for the broader ecosystem.

The application of new genomic technologies and methodologies is expected to generate knowledge about genes that trigger a more effective immune response in some species or lines; the effect that could be realised by editing these genes in more susceptible species or lines; potential lice attractants, repellents and assays; and the extent of additive genetic variation affecting the production and release of important immune factors and semiochemicals. Such knowledge could lead to the development of feed additives, gene edits, new vaccines and the enhancement of genomic breeding value estimation to promote host resistance. The epidemiological implications of these applications on the infectivity and virulence of aquatic diseases needs to be explored, and routines need to be devised to enhance the suppression of disease in the general aquaculture environment.

Such projects are ambitious in that it is hypothesised that specific semiochemical or immune pathways play major roles in differentiating disease‐resistant from disease‐susceptible hosts and that these differences are measurable, have a strong genetic basis, have implications for the epidemiology of infection and that genomic selection and/or gene editing approaches can be effectively and sustainably applied to reduce or eliminate the effect of disease on the host without counter evolutionary responses by the infectious agent taking effect. The long‐term suppression of disease will only be realised through a collaborative and coordinated multi‐disciplinary effort involving scientists working closely with aquaculture industry and government. Such efforts are likely to significantly advance our understanding of host–parasite and host‐disease interactions and mechanisms affecting resistance to disease and should result in significant economic impacts for aquaculture sectors, benefit the welfare of production animals and create ecosystem benefits for natural populations of these species.

## AUTHOR CONTRIBUTIONS

**Diego Robledo:** Conceptualization; funding acquisition; investigation; methodology; visualization; writing – original draft; writing – review and editing. **Lene Sveen:** Conceptualization; funding acquisition; investigation; methodology; visualization; writing – original draft; writing – review and editing. **Rose Ruiz Daniels:** Investigation; methodology; writing – original draft; writing – review and editing. **Aleksei Krasnov:** Conceptualization; funding acquisition; investigation; methodology; writing – original draft; writing – review and editing. **Andrew Lindsay Coates:** Investigation; methodology; writing – original draft; writing – review and editing. **Ye Hwa Jin:** Investigation; writing – original draft; writing – review and editing. **Luke Barrett:** Writing – original draft; writing – review and editing. **Marie Lillehammer:** Formal analysis; writing – original draft; writing – review and editing. **Anne Helena Kettunen:** Methodology; writing – original draft; writing – review and editing. **Ben L Phillips:** Conceptualization; funding acquisition; investigation; writing – original draft; writing – review and editing. **Tim Dempster:** Conceptualization; investigation; methodology; supervision; writing – original draft; writing – review and editing. **Andrea Doeschl‐Wilson:** Formal analysis; writing – original draft; writing – review and editing. **Francisca Samsing:** Writing – original draft; writing – review and editing. **Gareth Difford:** Investigation; writing – original draft; writing – review and editing. **Sarah Salisbury:** Investigation; writing – original draft; writing – review and editing. **Bjarne Gjerde:** Conceptualization; investigation; methodology; writing – original draft; writing – review and editing. **John‐Erik Haugen:** Investigation; writing – original draft; writing – review and editing. **Erik Burgerhout:** Methodology; writing – original draft; writing – review and editing. **Binyam Dagnachew:** Investigation; writing – original draft; writing – review and editing. **Dominic Kurian:** Investigation; writing – original draft; writing – review and editing. **Mark D. Fast:** Conceptualization; funding acquisition; methodology; visualization; writing – original draft; writing – review and editing. **Morten Rye:** Conceptualization; funding acquisition; writing – original draft; writing – review and editing. **Marcela Salazar:** Investigation; writing – original draft; writing – review and editing. **James Bron:** Conceptualization; funding acquisition; methodology; writing – original draft; writing – review and editing. **Sean Monaghan:** Investigation; writing – original draft; writing – review and editing. **Celeste Jacq:** Funding acquisition; investigation; writing – original draft; writing – review and editing. **Mike Birkett:** Funding acquisition; investigation; writing – original draft; writing – review and editing. **Howard Browman:** Funding acquisition; investigation; writing – original draft; writing – review and editing. **Anne Berit Skiftesvik:** Investigation; writing – original draft; writing – review and editing. **David Fields:** Investigation; writing – original draft; writing – review and editing. **Erik Selander:** Investigation; writing – original draft; writing – review and editing. **Samantha Bui:** Investigation; writing – original draft; writing – review and editing. **Anna Sonesson:** Writing – original draft; writing – review and editing. **Stanko Skugor:** Methodology; writing – original draft; writing – review and editing. **Tone‐Kari Knutsdatter Østbye:** Conceptualization; funding acquisition; investigation; methodology; writing – original draft; writing – review and editing. **Ross Houston:** Conceptualization; funding acquisition; investigation; supervision; writing – original draft; writing – review and editing.

## Data Availability

Data sharing not applicable ‐ no new data generated.
